# The Bacteroidetes *Aequorivita* sp. and *Kaistella jeonii* Produce Promiscuous Esterases With PET-Hydrolyzing Activity

**DOI:** 10.3389/fmicb.2021.803896

**Published:** 2022-01-05

**Authors:** Hongli Zhang, Pablo Perez-Garcia, Robert F. Dierkes, Violetta Applegate, Julia Schumacher, Cynthia Maria Chibani, Stefanie Sternagel, Lena Preuss, Sebastian Weigert, Christel Schmeisser, Dominik Danso, Juergen Pleiss, Alexandre Almeida, Birte Höcker, Steven J. Hallam, Ruth A. Schmitz, Sander H. J. Smits, Jennifer Chow, Wolfgang R. Streit

**Affiliations:** ^1^Department of Microbiology and Biotechnology, University of Hamburg, Hamburg, Germany; ^2^Molecular Microbiology, Institute for General Microbiology, Kiel University, Kiel, Germany; ^3^Center for Structural Studies, Heinrich-Heine-University, Düsseldorf, Germany; ^4^Department of Microbiology and Immunology, University of British Columbia, Vancouver, BC, Canada; ^5^Department of Biochemistry, University of Bayreuth, Bayreuth, Germany; ^6^Institute of Biochemistry and Technical Biochemistry, University of Stuttgart, Stuttgart, Germany; ^7^European Bioinformatics Institute (EMBL-EBI), Hinxton, United Kingdom; ^8^Wellcome Sanger Institute, Hinxton, United Kingdom; ^9^Graduate Program in Bioinformatics, University of British Columbia, Vancouver, BC, Canada; ^10^Genome Science and Technology Program, University of British Columbia, Vancouver, BC, Canada; ^11^Life Sciences Institute, University of British Columbia, Vancouver, BC, Canada; ^12^ECOSCOPE Training Program, University of British Columbia, Vancouver, BC, Canada; ^13^Institute of Biochemistry, Heinrich-Heine-University, Düsseldorf, Germany

**Keywords:** metagenomics, metagenomic screening, PET degradation, polyethylene terephthalate (PET), PETase, Bacteroidetes, Flavobacteriaceae

## Abstract

Certain members of the Actinobacteria and Proteobacteria are known to degrade polyethylene terephthalate (PET). Here, we describe the first functional PET-active enzymes from the Bacteroidetes phylum. Using a PETase-specific Hidden-Markov-Model- (HMM-) based search algorithm, we identified several PETase candidates from Flavobacteriaceae and Porphyromonadaceae. Among them, two promiscuous and cold-active esterases derived from *Aequorivita* sp. (PET27) and *Kaistella jeonii* (PET30) showed depolymerizing activity on polycaprolactone (PCL), amorphous PET foil and on the polyester polyurethane Impranil^®^ DLN. PET27 is a 37.8 kDa enzyme that released an average of 174.4 nmol terephthalic acid (TPA) after 120 h at 30°C from a 7 mg PET foil platelet in a 200 μl reaction volume, 38-times more than PET30 (37.4 kDa) released under the same conditions. The crystal structure of PET30 without its C-terminal Por-domain (PET30ΔPorC) was solved at 2.1 Å and displays high structural similarity to the *Is*PETase. PET30 shows a Phe-Met-Tyr substrate binding motif, which seems to be a unique feature, as *Is*PETase, LCC and PET2 all contain Tyr-Met-Trp binding residues, while PET27 possesses a Phe-Met-Trp motif that is identical to Cut190. Microscopic analyses showed that *K. jeonii* cells are indeed able to bind on and colonize PET surfaces after a few days of incubation. Homologs of PET27 and PET30 were detected in metagenomes, predominantly aquatic habitats, encompassing a wide range of different global climate zones and suggesting a hitherto unknown influence of this bacterial phylum on man-made polymer degradation.

## Introduction

PET is one of the major plastic pollutants found in landfills, oceans and other environments (Jambeck et al., 2015; Geyer et al., 2017). Our knowledge of microbial degradation of most plastics is rather limited, but recent research has demonstrated that some bacteria are able to degrade PET (Yoshida et al., 2016). Although it is unclear if larger crystalline fibers are degraded by bacteria, it is well known that some cutinases (EC 3.1.1.74), lipases (EC 3.1.1.3) and carboxylesterases (EC 3.1.1.1) can act on amorphous and low crystalline PET. These enzymes, often referred to as “PETases,” cleave the ester bond of the polymer to either produce bis-(2-hydroxyethyl) terephthalate (BHET), mono-hydroxyethyl terephthalate (MHET) or they complete degradation to terephthalic acid (TPA) and ethylene glycol (EG). TPA monomers can be further degraded via cleavage of the aromatic ring structure using known aryl pathways and can then enter the β-ketoadipate pathway (Wei and Zimmermann, 2017; Danso et al., 2019; Wright et al., 2021).

To date, only a limited number of bacterial and fungal species have been identified that are capable of breaking down PET to either its oligomers or monomers, TPA and EG. Most bacterial isolates with verified enzymatic PET-degrading activity are affiliated with the Gram-positive phylum Actinobacteria (Acero et al., 2011). The best characterized examples belong to the genera *Thermobifida* or *Thermomonospora* (Kleeberg et al., 1998; Chen et al., 2008; Hu et al., 2010; Acero et al., 2011; Ribitsch et al., 2012; Wei et al., 2014). Further, the leaf compost-derived cutinase LCC is closely related to Actinobacterial enzymes and is currently one of the best described and most active PETases (Sulaiman et al., 2012, 2014). Regarding Proteobacteria, the Gram-negative Betaproteobacterium *Ideonella sakaiensis* 201-F6 is capable of using amorphous PET as a major energy and carbon source (Yoshida et al., 2016). *I. sakaiensis’* genome also encodes a tannase which is designated MHETase as it is capable of degrading MHET. Besides, a number of other PETases affiliated with the Proteobacteria have been identified originating from e.g., *Pseudomonas aestusnigri* and *Vibrio gazogenes* (Ronkvist et al., 2009; Haernvall et al., 2017; Danso et al., 2018; Bollinger et al., 2020).

In a previous study, we identified potential PET esterases affiliated with the Bacteroidetes phylum using HMM profile database searches (Danso et al., 2018, 2019). These enzyme hits mainly occurred in metagenomes and genomes from marine environments and were annotated solely on the basis of homology. However, their enzymatic function and environmental distributions have not been studied within that framework, and we target these questions in the present study. Bacteroidetes representatives can be found in nearly all ecological niches including soils, oceans and fresh water and are part of the microbiome of many animals, especially as inhabitants of the intestinal tract (Wexler, 2007; Krieg et al., 2015; Hahnke et al., 2016; Munoz et al., 2016). The Bacteroidetes phylum, however, is highly heterogeneous and contains at least four classes of bacteria (e.g., Bacteroidia, Flavobacteria, Sphingobacteria, and Cytophagia), with each class having several thousand described species. The phylum contains non-spore forming and rod shaped microorganisms, some aerobic, but often anerobic, with an enormous metabolic diversity (Krieg et al., 2015). The global distribution of Bacteroidetes representatives is likely due to their ability to decompose a very wide variety of bio-based polymers such as cellulose, chitin or algal cell walls. In particular, the decomposition of polysaccharides (cellulose and hemicellulose) by Bacteroidetes inhabiting the intestinal tract of humans and animals has been well-studied in gut microbiome research (Thomas et al., 2011).

Here, we provide the first experimental evidence that different Bacteroidetes representatives have evolved promiscuous esterases that degrade the PET polymer. We show that at least two Bacteroidetes genera, *Aequorivita* and *Kaistella* (formerly *Chryseobacterium*), harbor PET-active enzymes and elucidated the crystal structure of PET30. These enzymes have relatively low turnover rates, indicating that PET hydrolysis may be a side reaction. Still, given their abundance and diversity, we speculate that the described bacteroidetal PET-active enzymes could have considerable impact on long-term degradation of PET in the marine environment.

## Materials and Methods

### Bacterial Strains, Plasmids, and Primers

Bacterial strains, plasmids and primers used in this study are listed in Supplementary Tables 1, 2. If not mentioned otherwise, *Escherichia coli* clones were grown in LB medium (1% tryptone/peptone, 0.5% yeast extract, 1% NaCl) supplemented with appropriate antibiotics (25 μg/ml kanamycin, or 100 μg/ml ampicillin) at 37°C for 18 h.

### Databases Used in This Study and Bioinformatic Analyses

Nucleotide and amino acid sequences of putative and confirmed PETases were acquired from databases integrated into the NCBI^1^, UniProt^2^ and IMG (JGI)^3^ servers (Markowitz et al., 2012; NCBI Resource Coordinators, 2017; The UniProt Consortium, 2017). Human gut sequences were retrieved from the Unified Human Gastrointestinal Protein (UHGP) catalog (PMID: 32690973). Sequences were compared to others deposited in the NCBI databases using BLAST alignment tools (Agarwala et al., 2016). Amino acid sequence HMM search was carried out using the HMMER^4^ webpage or a local version of the software (v3.1b2) (Mistry et al., 2013) with downloaded datasets. Structural information on the enzymes was retrieved from the RCSB-PDB (Berman et al., 2000) database.

Sequence data were processed and analyzed using ChromasPro 2.1.8 (Technelysium, Brisbaine Australia) or SnapGene (GSL Biotech LLC, San Diego CA, United States). Amino acid alignment was constructed using structural alignments with T-Coffee (Notredame et al., 2000) and was further visualized with Bioedit (Hall, 1999). The model structures of bacteroidetal PETase-candidates were modeled with the Robetta server (Kim et al., 2004) using the *Is*PETase crystal structure (6EQE) as a backbone. A phylogenetic tree was constructed using the RAxML-NG autoMRE algorithm (Kozlov et al., 2019) with the treesapp create command implemented in TreeSAPP (Morgan-Lang et al., 2020) with maximum bootstraps set at 1,000. RAxML-MG has recently been shown to return the best scoring tree for highest number of datasets when compared against other fast maximum likelihood (ML) methods (Kozlov et al., 2019), allowing a large number of maximum bootstraps to be used to produce as conservative a tree as possible. Sequences were assigned NCBI lineages according to source organisms listed in Table 1 and Supplementary Table 3, and colors were assigned to the tree at the phylum level using the treesapp color command. The tree was visualized in iTOL (Letunic and Bork, 2019).

**TABLE 1:** Key traits of predicted bacteroidetal PET esterases.

Predicted PETase	GenBank entry/MGY identifier	Phylogenetic Affiliation	aa/MW (kDa)	Derived from	Expression level/solubility	Active on						
						*p*NP-C6/-C10	TBT	Impranil^®^ DLN	PCL	BHET	PET-foil	PET particles
PET27	WP_111881932	*Aequorivita* sp. *CIP111184*	364/37.8	Antarctic source (Li et al., 2017)	High/majority in inclusion bodies	+	+	+	+	+	+	+
PET28	WP_073216622	*Aequorivita viscosa*	365/38.3	Seaweed (Li et al., 2017)	High/majority in inclusion bodies	+	+	+	+	+	-	-
PET29	WP_052671284	*Aequorivita vladivostokensis*	365/39.3	Troitsa bay, Sea of Japan (Li et al., 2017)	High/majority in inclusion bodies	+	+	+	+	+	-	-
PET30	WP_039353427	*Kaistella jeonii*	366/37.4	Antarctic moss (Li et al., 2017)	High/majority soluble	+	+	+	+	+	+	+
PET38	WP_083800582.1/GCA_ 000194605.1	*Fluviicola taffensis*	447/40.4	River, United Kingdom (Woyke et al., 2011)	Low	-	-	N.D.	-	-	-	-
PET53	k99_709705_13	*Aequorivita* sp.	294/37.8	Marine aquaculture fish tank metagenome/unpublished data University of Hamburg	Low	*-*	*-*	N.D.	*-*	N.D.	N.D.	N.D.
PET57	GUT_GENOME137663_00143	*Porphyromonas* sp.	323/36.3	Human gut (Mitchell et al., 2019; Almeida et al., 2021)	High/majority soluble	-	-	+	+	N.D.	N.D.	N.D.
PET58	GUT_GENOME065712_01381	*Porphyromonas bennonis*	338/37.6	Human gut (Mitchell et al., 2019; Almeida et al., 2021)	High/majority in inclusion bodies	-	-	-	+	N.D.	N.D.	N.D.
PET59	GUT_GENOME243617_00165	*Porphyromonas* sp.	345/38.4	Human gut (Mitchell et al., 2019; Almeida et al., 2021)	High/majority soluble	-		-	+	N.D.	N.D.	N.D.

*TBT, tributyrin; BHET, bis-(2-hydroxyethyl) terephthalate; PCL, polycaprolactonate; pNP-C6/C10, para-nitrophenyl esters with chain length C6 or C10; aa, amino acids; MW, molecular weight. N.D. not determined. +, active; -, not active.*

*PET57-59 were extracted from the gut genomes available at: https://www.ebi.ac.uk/metagenomics/genomes/MGYG-HGUT-01059 (PET57); https://www.ebi.ac.uk/metagenomics/genomes/MGYG-HGUT-01060 (PET58) and https://www.ebi.ac.uk/metagenomics/genomes/MGYG-HGUT-00764 (PET59).*

**FIGURE 1:**
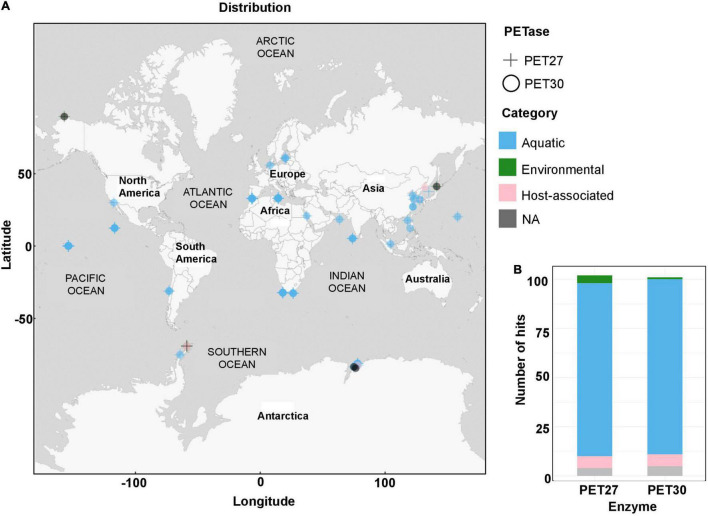
Global distribution of PET27 and PET30 homologs. **(A)** PET27 and PET30 homologs containing metagenomes were visualized on a world map containing circles for the different metagenomes. The cut-off in the similarity searches was set to 50%. Data depicted include only hits to bacteria affiliated with the Flavobacteria-Cytophaga-Bacteroidetes (FCB) group. The metagenomes searched and included in this figure are listed in Supplementary Table 2. **(B)** Number of hits observed in the same global metagenomes. Color code indicates the type of habitat: air, aquatic, terrestrial host-associated and/or engineered.

Scanning IMG/M was completed on 19/November/2020 for PET30 and on 14/January/2021 for PET27. Geo locations were used as provided whenever available. In case no Geo location was available, whenever possible, information about isolation source/location/city/country were used to look up Geo coordinates on GeoHack.^5^ The map representing the frequency and geographical distribution of PET hydrolases in metagenomes (Figure 1) was constructed using QGis Desktop 2.18.5^6^.

### Heterologous Expression of Putative Polyethylene Terephthalate Esterase Genes in *Escherichia coli* BL21 (DE3)

The putative PETases were extracted from metagenomic datasets (Table 1), therefore the gene sequences were optimized for expression in *E. coli* and synthesized into pET21a(+) vector at Biomatik (Wilmington, United States). The obtained constructs were sequenced at Microsynth Seqlab GmbH (Goettingen, Germany) and checked for correctness by comparing to the original sequences. *E. coli* T7-Shuffle or *E. coli* BL21 (DE3) cells were used for heterologous expression of possible PETases. The *Is*PETase gene in pMAL-p4x was expressed in *E. coli* BL21 and purified by its maltose-binding tag. The cultures were grown aerobically in auto-induction medium (ZYM-5052) (Studier, 2005) containing 100 μg/ml ampicillin at 37°C until they reached an OD_600_ of 1.0. The proteins were expressed afterward at 22°C for 16–20 h. The cells were harvested and lysed with pressure using a French press. Afterward, the proteins harboring a sixfold C-terminal histidine tag were purified with nickel-ion affinity chromatography using Ni-NTA agarose (Qiagen, Hilden, Germany) and analyzed by SDS-PAGE. The elution buffer was exchanged against 0.1 mM potassium phosphate buffer pH 8.0 in a 10 kDa Amicon Tube (GE Health Care, Solingen, Germany).

### Biochemical Characterization of PET27 and PET30

For activity tests, both enzymes were assayed using purified recombinant protein. bis-(2-hydroxyethyl) terephthalate (BHET) and polycaprolactone (PCL) agar plates were prepared as described elsewhere (Pérez-García et al., 2021). The polyester polyurethane Impranil DLN containing LB agar plates were prepared according to Molitor et al. (2020) with LB medium. For the *p*NP-assay, unless otherwise indicated, a total amount of 0.1–1 μg of the enzymes were added to a substrate solution containing 190 μl of either 0.2 M sodium phosphate buffer or 0.1 M potassium phosphate with a defined pH between 7 and 8 and 10 μl of 0.1 mM *p*NP-substrate dissolved in isopropanol. After incubating the samples for 10 min, the assay was stopped by adding 200 mM of Na_2_CO_3_. Afterward, the samples were centrifuged at 4°C, 13,000 rpm for 3 min. As substrates, *p*NP-esters with chain lengths of C4, C6, C8, C10, C12, C14, C16 and C18 were tested. After incubation at defined temperatures, the color change from colorless to yellow was measured at 405 nm in a plate reader (Biotek, Winooski, United States). All samples were measured in triplicate. To determine the optimal temperature, samples were incubated between 10 and 90°C for 10 min. The influence of pH conditions on the activity of each enzyme was measured in citrate phosphate (pH 3.0, 4.0, and 5.0), potassium phosphate (pH 6.0, 7.0, and 8.0) and carbonate bicarbonate buffer (pH 9.2 and 10.2). The impact of cofactors, solvents, detergents, and inhibitors was assayed at different concentration levels. The possible cofactors Ca^2+^, Co^2+^, Cu^2+^, Fe^3+^, Mg^2+^, Mn^2+^, Rb^2+^, and Zn^2+^ with a final concentration of 1 and 10 mM were used. Detergent stability was assayed with sodium dodecyl sulfate (SDS), Triton X-100 and Tween 80 at 1 and 5% (w/v, v/v) concentration. The inhibitory effect of ethylenediaminetetraacetic acid (EDTA), dithiothreitol (DTT) and phenylmethanesulfonyl fluoride (PMSF) was tested at 1 and 10 mM concentration. After 1 h incubation in the presence of these substances, the residual activity was determined after 10 min incubation at the optimal temperature with *para-*nitrophenol- (*p*NP-) C6 and at the optimal pH.

For the verification of enzymatic PET hydrolysis, a 7 mg platelet (Ø 5 mm) of low-crystallinity PET film (Goodfellow GmbH, Bad Nauheim, Germany), which corresponds to 36.4 μmol of the terephthalic acid-ethylene glycol (TPA-EG) unit, was folded in half and used as substrate together with 200 μg of enzyme in 200 μl of 100 mM potassium phosphate buffer at pH 8.0. Incubation was carried out under continuous shaking at 400 rpm in 1.5 ml microcentrifuge tubes at 30°C, if not stated otherwise.

Analysis of breakdown products was performed with an UltiMate™ 3000 UHPLC system from Thermo Fisher Scientific (Waltham, MA, United States) using a Triart C18 column (YMC Europe GmbH, Dinslaken, Germany) with a dimension of 100 × 2.0 mm containing particles with 1.9 μm diameter. Isocratic elution was performed using a mobile phase consisting of 20:80 (v/v) acetonitrile and water (acidified with 0.1% vol trifluoroacetic acid) at a flowrate of 0.4 ml min^–1^. UHPLC samples were prepared by mixing 50 μl of incubation supernatant with 200 μl acetonitrile (acidified with 1% vol trifluoroacetic acid), followed by centrifugation at 10,000 × g for 3 min and transferring 200 μl of the supernatant into 600 μl water. Fifteen microliter of sample were injected per measurement and detection was performed at 254 nm with a VWD-3400 detector from Thermo Scientific (Waltham, MA, United States). The UHPLC profiles were plotted and edited using the software MATLAB version R2020b [The MathWorks, Inc., Natick, MA, United States (Matlab, 2012)]. Quantification of peak areas was performed using data analysis software supplied with the Compass HyStar software package from Bruker (Billerica, MA, United States).

### Crystallization and Data Collection

Crystallization of PET30ΔPorC was achieved by sitting-drop vapor-diffusion at 12°C. 0.2 μl of 10.2 mg/ml PET30ΔPorC in 100 mM phosphate buffer pH 8.0 and 0.1 μl reservoir solution consisting of 0.1 M sodium aetate pH 4.6 and 25% (w/v) PEG 4000 were mixed. This drop was equilibrated against reservoir solution and crystals formed after several weeks. Crystallization drops were overlayed with mineral oil and the crystals were dragged through it for cryoprotection, flash frozen and diffraction data were collected at beamline P13 (DESY, Hamburg, Germany). The PET30ΔPorC crystals had the space group P 43 21 2 and diffracted to 2.1 Å resolution.

### Structure Determination

A complete data set of the PET30ΔPorC was collected at beamline P13 (DESY, EMBL, Hamburg, Germany) at 100 K and wavelength 0.9795 Å up to 2.1 Å resolution. All data were processed using the automated pipeline at the EMBL HAMBURG and reprocessed afterward using XDS (Kabsch, 2014). The above obtained model for PET30ΔPorC by TOPMODEL was successfully used to phase the 2.1 Å data set of PET30ΔPorC using the PHASER program from the PHENIX program suite (Afonine et al., 2012; Mulnaes et al., 2020). The structure was then refined in iterative cycles of manual building and refinement in coot followed by software-based refinements using the program suite Phenix (Emsley and Cowtan, 2004; Liebschner et al., 2019). All residues were in the preferred and additionally allowed regions of the Ramachandran plot. The data collection and refinement statistics are listed in Supplementary Table 4. The images of the models were prepared using PyMOL (DeLano, 2002) and UCSF Chimera X.^7^ The structure was deposited at the worldwide protein data bank under the accession code 7PZJ.

### Confocal Laser Scanning Microscopy of Polyethylene Terephthalate Foil Platelets

The starter culture of *K. jeonii* was grown in R2A medium at 22°C and 130 rpm to a cell density of 0.2. 1% of the starter culture was inoculated into 30 ml fresh R2A medium and PET foil platelets were put into the cultures. PET platelets were removed after 5-7 days, washed three times with PBS and subsequently given into μ-Slide 8 wells plates from ibidi GmbH (Martinsried, Germany). Cells were stained using 100 μl of LIVE/DEAD stain BacLight Viability Kit (Thermo Fisher Scientific). The stain is composed of propidium iodide (PI) dying dead cells with a damaged membrane and causing red fluorescence and green fluorescence SYTO 9™ dying all bacterial membranes of living cells. Therefore, 10 μl PI and 10 μl SYTO 9™ were mixed. 15 μl of the nucleic acid-binding stains were pipetted into 5 ml PBS. The PET platelets were incubated for 1 h in the dark at room temperature. Afterward, the samples were investigated under the microscope Axio Observer Z1/7, LSM 800 using objective C-Apochromat 63x/1.20 W Korr UV VisIR (both Carl Zeiss Microscopy GmbH, Jena, Germany) using the Channels Syto 09 (528/20 nm emission wavelength) and PI (645/20 nm emission wavelength).

## Results

### Profile Hidden Markov Model Searches Identify Potential Bacteroidetal PETases

Protein sequences from both genomes and metagenomes were screened using the previously described Hidden Markov Model (HMM) (Danso et al., 2018) to enrich the diversity of PET-active enzymes from Bacteroidetes. The global searches were performed in publicly available datasets of NCBI GenBank and additionally in several private datasets harboring human-associated and environmental Bacteroidetes sequences (Table 1). Searches were conducted from January until March 2019. This global search initially resulted in the identification of 37 potential PETase sequences from Bacteroidetes with a bit score above 298.7. After sequence comparison, nine distinct hits were chosen. These candidates belonged to bacteroidetal genomes originating from either Seaweed (Li et al., 2017), Antarctic moss (Li et al., 2017), river sediment (Woyke et al., 2011), an aquaculture (own unpublished dataset) or human gut microbiomes (Mitchell et al., 2019; Almeida et al., 2021; Table 1). Most of these candidates were affiliated with the Flavobacteriaceae genus *Aequorivita* sp. (PET27-29, PET31 and PET53). PET29 and PET31 were highly similar on amino acid level (< 98% identity) but differed in the length of their sequence by 10 amino acids (aa). PET30, annotated as a potential lipase, was derived from the published genome sequence of *Kaistella jeonii* NCTC 13459. The predicted PETases PET57-59 were derived from bacteria affiliated with the genus *Porphyromonas* sp. (Porphyromonadaceae), while the predicted enzyme PET38 was derived from the species *Fluviicola taffensis* (Cryomorphaceae).

### Recombinant PET27 and PET30 Hydrolyze Polycaprolactone, Impranil®-DLN and Polyethylene Terephthalate Foil

**FIGURE 2:**
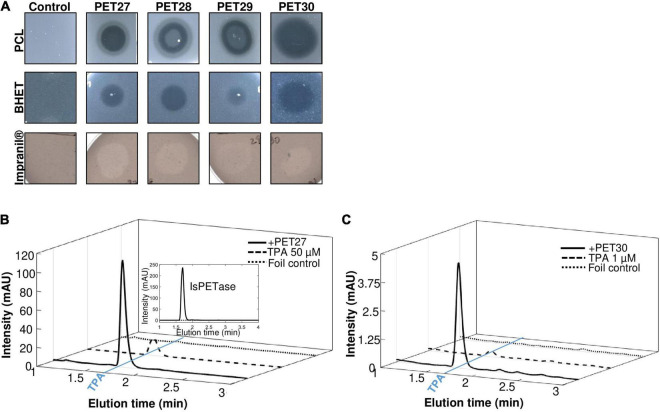
Hydrolytic activities of PET27-PET30 on PCL, BHET, Impranil^®^ DLN and PET foil. Activities on PCL, BHET and Impranil^®^ DLN were visible by halo-formation on agar plates **(A)**. 10 μl of purified enzyme (10-100 μg) were applied to agar plates containing either 500 mg/l PCL, 5 mM BHET or 1% Impranil^®^ DLN. Clearing zones were observed after 12 h at 30°C. Control indicates plates without enzymes but treated with 10 μl buffer. UHPLC profiles of PET27 **(B)** and PET30 **(C)** after incubation on PET foil for 120 h. Two hundred microliter of recombinant and purified enzymes (1 mg/ml) were applied to amorphous foil and incubated over 120 h at 30°C. The *Is*PETase was included for reasons of benchmarking in **(B)** at 1 mg/assay (upper right corner). Graphs shown are representative graphs and were repeated at least three times.

The nine candidate genes of the predicted PETases were synthesized, cloned into the expression vector pET21a(+) (Biomatik, Wilmington, DA, United States) and transformed in *E. coli* BL21 and T7-Shuffle cells (Supplementary Table 1). Initial tests using recombinant purified proteins and tributyrin (TBT)-containing agar plates indicated that the genes PET27-30 coded for active esterases. The remaining enzymes PET38, PET53, PET57, and PET58 were inactive and were either produced as insoluble proteins and/or only at very low amounts (Table 1). Because of these obvious difficulties facing their expression, these four predicted enzymes were not further characterized. Additional tests with PET27-PET30 indicated that these enzymes hydrolyzed the esters *p*NP-hexanoate (C6), and *p*NP-decanoate (C10, Table 1). All four recombinant enzymes were able to hydrolyze the polymeric polycaprolactone (PCL), the PET-constituent BHET, and the polyester polyurethane Impranil^®^ DLN (Covestro AG, Leverkusen, Germany) (Table 1 and Figure 2A). The enzymes produced clear halos on agar plates containing these substrates after overnight incubation at 30°C (Figure 2A and Table 1). PET-hydrolyzing activities were confirmed for the enzymes PET27 and PET30 on amorphous PET foil as substrate in a 200 μl reaction volume by UHPLC analyses. In these tests, 1 mg ml^–1^ PET27 released 871.8 ± 200.4 μM (corresponds to 174.4 ± 40.0 nmol in 200 μl reaction volume) of TPA in 120 h at 30°C from a 7 mg PET platelet. PET foil (7 mg) corresponds to 36.4 μmol of a TPA-EG monomer unit (Figure 2B and Table 2). Surprisingly, under the same conditions, PET30 released only 15.9 ± 9.5 μM TPA (corresponds to 3.2 ± 1.9 nmol; Figure 2C and Table 2). These results were directly benchmarked with recombinant *Is*PETase, of which 1 mg ml^–1^ released under the same conditions 4,055.7 ± 516.9 μM of TPA (corresponds to 811.1 ± 103.4 nmol). Thus, *Is*PETase is 4.7-fold more active compared to PET27 and approximately 253-fold more active compared to PET30. Because of the relatively low turnover rates observed for PET27 and PET30 on PET foil, it can be assumed that PET is not the preferred substrate of both enzymes.

**TABLE 2:** Amount of TPA released by different PET active enzymes.

Enzyme	Released TPA-EG unit			Av. weight loss of PET foil [%]
	[μM]	[nmol]	[μg]	
PET27	871.8 ± 200.4	174.4 ± 40.0	33.5 ± 7.7	0.45
PET30	15.9 ± 9.5	3.2 ± 1.9	0.6 ± 0.3	0.01
PET30ΔPorC	23.3 ± 9.2	4.7 ± 1.8	0.9 ± 0.3	0.01
*Is*PETase	4,055.7 ± 516.9	811.1 ± 103.4	155.8 ± 19.9	2.23

*The different recombinant and purified enzymes were incubated at a concentration of 1 mg ml^–1^ for a time period of 120 h at 30°C. For the tests a circular piece of 7 mg PET foil which corresponds to 36.4 μmol of the TPA-EG unit (Ø 5 mm, and as specified in section “Materials and Methods”) was employed and folded once in the middle. Incubations were carried out in a reaction volume of 200 μl. Data are mean values with standard deviations of a minimum of 3 and up to 6 measurements per sample.*

### Biochemical Characterization and Activity of PET27 and PET30 on Esterase Substrates

**FIGURE 3:**
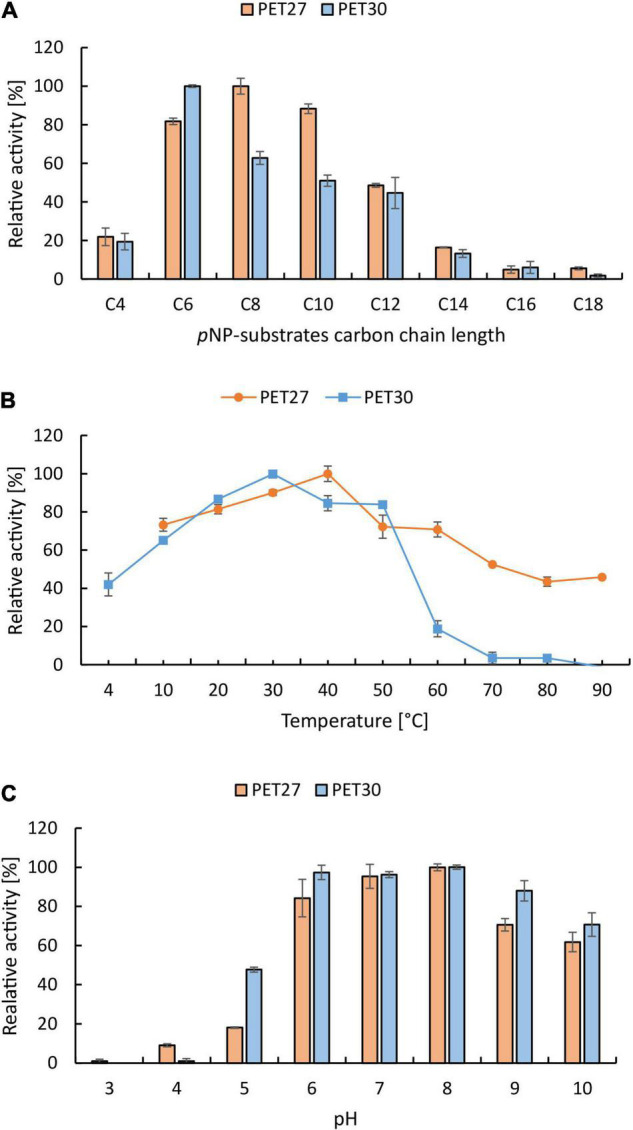
Biochemical characterization of PET27 and PET30 using *p*NP-substrates. Data represent mean values of at least three independent samples. Substrate preference **(A)** was tested with *p*NP-butyrate (-C4) to –stearate (-C18). Temperatures **(B)** and pH **(C)** were tested with *p*NP-octanoate (-C8) for PET27 and with *p*NP-hexanoate (-C6) for PET30. All assays except B were conducted at 40°C for PET27 and at 30°C for PET30. Both enzymes have their highest activity at cold to moderate temperatures **(B)** and at slightly acidic to alkaline pH **(C)**.

Both recombinant enzymes were characterized in more detail with *p*NP-esters. A substrate spectrum was recorded with *p*NP-esters, which had acyl chain lengths of 4–18 C-atoms. PET27 and PET30 revealed a relatively narrow spectrum of substrates they could hydrolyze. The highest activities were observed with *p*NP-hexanoate (-C6) for PET30 and *p*NP-octanoate (-C8) for PET27 (Figure 3A). The optimal temperature of PET30 is 30°C, but 80% activity was observed at 20°C and between 40 and 50°C (Figure 3B). In contrast to that, PET27 shows a better activity at higher temperatures with an optimum at 40°C and even 45% activity at 90°C. Surprisingly, at 10°C, both enzymes still showed a relative activity of 65% (PET30) and 73% (PET27). PET30 remained active at 4°C showing a relative activity of 42% on *p*NP-C6. Concerning the optimal pH, PET27 was most active between pH 7–8 and PET30 between pH 6-8 when tested in 0.1 M potassium phosphate (Figure 3C).

To further characterize the effects of metal ions, different ions (Ca^2+^, Co^2+^, Cu^2+^, Fe^3+^, Mg^2+^, Mn^2+^, Ni^2+^, and Zn^2+^) were added to the assays at 1 and 10 mM final concentrations. Metal ions have a minor influence on PET27. Addition of Ca^2+^ resulted in a 1.4-fold increase of the activity (Supplementary Figure 1A). In case of PET30, addition of Zn^2+^, Ni^2+^, and Co^2+^ resulted in an up to threefold increase of activity.

The kinetic parameters for PET27 and PET30 were determined with *p*NP-C6 at 30°C and pH 8 according to Michaelis-Menten. Thereby, PET27 revealed a *v*_*max*_ of 4.9 nmol min^–1^, a *k*_*cat*_ of 19.08 s^–1^, a *K*_*m*_ of 1.37 mM and a *k*_*cat*_/*K*_*m*_ value of 13,859.27 M^–1^ s^–1^. For PET30, we calculated a *v*_*max*_ of 2.3 nmol min^–1^, a *k*_*cat*_ of 8.9 s^–1^, a *K*_*m*_ of 0.3 mM and a *k*_*cat*_/*K*_*m*_ value of 26,136.11 M^–1^ s^–1^.

Further, PET30 was investigated in more detail. To assess thermostability, the enzyme was incubated at 50 and 60°C for 3 h, after which the enzyme retained only 23 and 5% of its original activity, respectively (Supplementary Figure 1B). As inhibiting substances, EDTA, DTT and PMSF were applied in final concentrations of 1 and 10 mM (Supplementary Figure 1C). The presence of DTT and PMSF (1 and 10 mM) inactivated PET30 almost completely, whereas EDTA at 1 and 10 mM had no large impact on the enzyme’s activity. A concentration of 1 and 5 % of the detergents Triton X-100, Tween 80 and SDS decreased PET30’s activities (Supplementary Figure 1D).

As both enzymes were active at lower temperatures, PET foil degradation was assayed at 4°C. Over a time of 30 days in a 200 μl reaction volume, TPA release was measured. Under these conditions, 1 mg ml^–1^ of PET30 released an average of 6.1 μM of TPA (corresponds to 1.2 nmol). Interestingly, *Is*PETase released under the same conditions a similar amount (5.9 μM TPA corresponds to 1.2 nmol). Notably, under these conditions, no detectable amounts of TPA were released with PET27 after 30 days.

### Amino Acid Sequence and Structural Analyses Identify Unique Traits of Bacteroidetal Polyethylene Terephthalate-Hydrolyzing Enzymes

While all four enzymes PET27-PET30 were able to hydrolyze PCL, BHET and Impranil^®^ DLN, only PET27 and PET30 were able to depolymerize PET. To identify the key differences that confer this activity on PET, all predicted PETases were studied on sequence and structural level. With an average of 330 aa, the predicted molecular weights of the enzymes ranged from 36 to 48 kDa. Each candidate contained a C-terminal signal domain for protein transport to the periplasm as predicted with SignalP 5.0 (Almagro Armenteros et al., 2019), supporting the notion that these are secreted proteins (Table 3). Remarkably, the predicted PETases PET27-PET30 and PET38 showed a type IX secretion system (T9SS)/PorC-type sorting domain-containing part at the C-terminus. It has been described earlier by a profile HMM from the TIGRFAM database (TIGR04183). T9SS sorting domains are involved in protein transport across the bacterial outer membrane and have so far been described as a Bacteroidetes-specific secretion system (Sato et al., 2010; Shoji et al., 2011; de Diego et al., 2016). The predicted domain encompassed 62–64 aa in the cases of PET27-PET30 and PET38. PET57 and 58 carried truncated sorting domains of 42 and 55 aa in length. This observation also implies that these enzymes are most likely exoenzymes (Table 3 and Supplementary Figures 3, 4). To ensure that this C-terminus does not affect catalytic activity, a deletion mutant designated PET30ΔPorC was created that lacked the sorting sequence between the amino acids 300–366. Activity tests confirmed that it was not affected in its activities using *p*NP-C6 or PET foil (Supplementary Figure 1E and Table 2). The enzyme released similar amounts of TPA as it was observed for the native PET30 (Table 2).

**TABLE 3:** Conserved motifs and structural features identified in the predicted bacteroidetal PET-hydrolyzing esterases.

Enzyme	N-terminus			Catalytic triad	Substrate binding site	Disulf. bridge*	C-terminus			
	Aln 1st aa	Length [N]	SP cleavage site				Aln last aa	Length [N]	Secondary structure	CD
*Is*PETase	A47	47	27–28	D-H-S	Y-M-W	2x	C273	18	N/A	N/A
LCC	D53	53	21–22	D-H-S	Y-M-W	1x	L274	19	N/A	N/A
Cut190	R64	64	N/A	D-H-S	F-M-W	2x	L278	29	N/A	N/A
PET27	P36	36	23–24	D-H-S	F-M-W	1x	L265	99	7xβ	PorC
PET28	P36	36	23–24	D-H-S	F-M-W	1x	L265	100	6xβ	PorC
PET29	P36	36	23–24	D-H-S	F-M-W	1x	L265	100	α, 4xβ, α, 2xβ	PorC
PET30	P36	36	23–24	D-H-S	F-M-Y	1x	A266	100	7xβ	PorC
PET38	S7	7	19–20	D-H-S	F-M-A	1x	I279	168	5xβ, α, 2xβ	PorC
PET53	T36	36	22–23	D-H-S	F-M-W	1x	V268	86	4xβ	N/A
PET57	N35	35	25–26	D-H-S	W-M-Y	N/A	F289	34	α + loops	N/A
PET58	I34	34	24–25	D-H-S	F-M-Y	N/A	F293	45	loops + α	N/A
PET59	Y48	48	24–25	D-H-S	F-M-Y	N/A	Y294	51	α + semi-α	N/A

*The Ideonella sakaiensis PETase (IsPETase, PDB: 6EQE; Yoshida et al., 2016; Austin et al., 2018), the LCC (4EB0; Sulaiman et al., 2014) and the Cut190 (4WFI; Miyakawa et al., 2015) were included for benchmarking purposes.*

Aln: Alignment; SP: Signal Peptide; α, α -helix; ß, ß-sheet; N/A, not identified; *, verified and predicted disulfide bonds; CD: Conserved Domain; PorC, Por secretion system C-terminal sorting domain.

**FIGURE 4:**
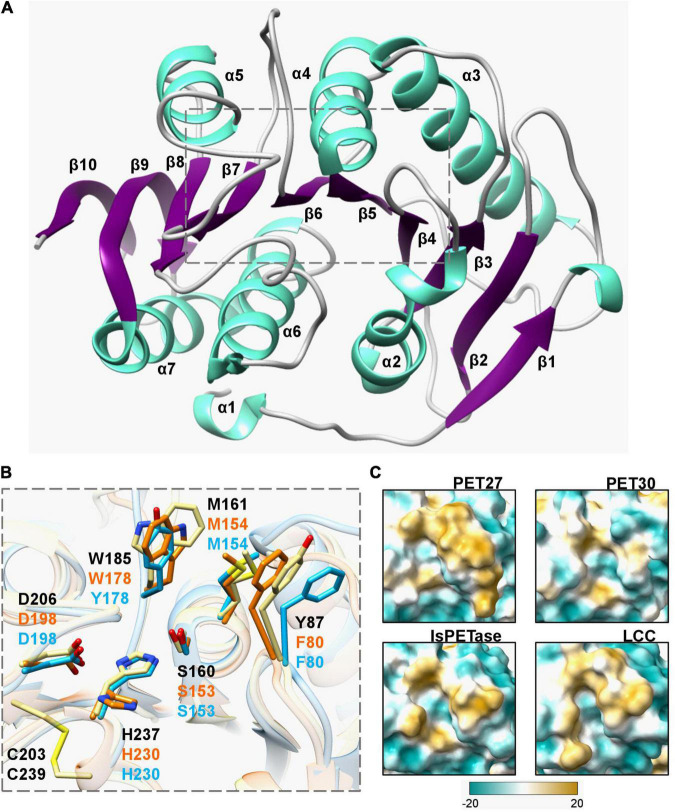
Crystal structure of the PET-hydrolyzing bacteroidetal PET30ΔPorC including active site and hydrophobicity comparison. **(A)** Overall structure of PET30ΔPorC. The structure was solved by X-ray crystallography to a resolution of 2.1 Å and is shown as cartoon representation. Helices are colored in light turquoise and the beta-sheets are shown in purple. The number of the secondary structure elements are numbered according to the occurrence in the sequence. **(B)** Comparison of active site residues. All three enzymes PET30ΔPorC (light blue), PET27 (orange) and *Is*PETase (light yellow) have the typical residues of Ser-hydrolases at the catalytically active positions (Ser, His, and Asp), but PET27 and PET30ΔPorC differ in some of the amino acids associated with PET-binding. The residues of *Is*PETase are indicated in black. PET30 and PET27 lack a disulfide bridge in the proximity of a catalytic loop. Supplementary Figure 4 provides the positions of these residues in details on the amino acids level. The 3D structure of PET27 was modeled using the Robetta server (Kim et al., 2004) using the *Is*PETase crystal structure (6EQE) as a backbone. **(C)** Surface hydrophobicity around the tunnel leading to the active site of four PET-degrading enzymes. Hydrophilic regions are displayed in turquoise and hydrophobic in gold.

Further analyses of the amino acid sequences identified a G-x-S-x-G motif which is typical for α/β serine hydrolases (Ollis et al., 1992) and a catalytic triad that consists of the residues Asp-His-Ser (Figure 4 and Supplementary Figure 4). Potential substrate binding sites in all bacteroidetal enzymes were identified containing the aa Phe-Met-(Trp/Tyr/Ala). The latter differed from the known *Is*PETase, the LCC and PET2 binding sites, in which a Tyr-Met-Trp motif is present (Table 3). PET57 is the only exception with a Trp-Met-Tyr binding site. PET27, however, has the Tyr replaced with a Phe that is identical to Cut190, while PET30 has in addition the Trp in position 3 replaced with a Tyr (Table 3). This single amino acid substitution in the predicted substrate binding pocket of PET30, however, is not solely responsible for PET-degrading activity. The mutants PET30_F80Y and PET30_Y178W as well as a version containing both of these point mutations even lost BHET- and PET-degrading activity (data can be shown upon request).

### Structure Determination of PET30 and Structural Modeling of the Other Putative PETases

For structural insights into the mechanism of polymer degradation, the structures of all predicted PETases, except PET30, were modeled using the *Is*PETase (PDB code 6EQE) as template. Crystallization was conducted with purified PET30ΔPorC and X-ray structure determination using a model of PET30 for molecular replacement. The structure was solved at 2.1 Å resolution, containing one monomer in the asymmetric unit, with 16.2% for R_*work*_ and 21.9% for R_*free*_. Data collection and structure refinement statistics are given in Supplementary Table 4. Coordinates of PET30ΔPorC were deposited under the PDB accession code 7PZJ. The PET30ΔPorC protein shows a canonical α/β-fold consisting of a central twisted β-sheet composed of 9 β-strands flanked by 7 α-helices on both sides (Figure 4A), as already reported for homologous structures, i.e., PETases and cutinases (Han et al., 2017; Numoto et al., 2018). The structure of PET30ΔPorC revealed an extra β-sheet (β10) which is located at the C-terminus and connects the PorC domain which has been deleted in this construct. PET30ΔPorC represents the overall fold of several PETases as revealed by a similarity search performed by PDBeFold.^8^ Here, especially the known structures of the PETase from *Ideonella sakaiensis* (for example PDB code 5XH3; Han et al., 2017), show high consistency indicated by the rmsd of 1.3-1.5 Å. Also, high structural similarity can be seen with the structure of PE-H from *Pseudomonas aestusnigri* (PDB code 6SCD rmsd 1.3) and the cutinase from a member of the Burkhoderiales bacteria family (PDB code 7CWQ, rmsd of 1.32 (Bollinger et al., 2020; Chen et al., 2021). One disulfide bond is present in PET30ΔPorC (C262-C285), a common feature for Type II PET-degrading enzymes (Joo et al., 2018). In the PETase from *I. sakaiensis* (PDB code 5YNS), a second disulfide bond is present which is located closely to the active site. Mutational analysis revealed that this disulfide bond plays a crucial role in activity since mutation of the involved cysteine to serine completely abolished activity, likely due to a destabilizing effect on the active site. The sequence in PET30ΔPorC at this position deviates, and here, G195 and V232 are present. In the PE-H structure, also no disulfide bridge can be found, although one cysteine residue remained (G195 and C251). From the many structures of the PETase from *I. sakaiensis*, one was solved in complex with 2-hydroxyethyl methyl terephthalate (HEMT; PDB code 5XH3). Here, HEMT is bound via interactions with W156, I179, H208, A131, W130, Y58, and M132. We looked into the active site and overlaid the HEMT molecule with our PET30ΔPorC structure (Supplementary Figure 2). Here similar interactions of the HEMT molecule by the PET30ΔPorC protein can be deduced mediated by Y178, T200, H230, W152, F80, and M154 (Figure 4B).

The largest differences can be seen in the C-terminal part affiliated with the T9SS-domain. It differed largely from the *Is*PETase and consisted of up to seven predicted β-sheets and, occasionally, a few α-helices (Supplementary Figure 3A and Table 3). Another difference between PET27, PET30, *Is*PETase and LCC is the surface hydrophobicity of the region channeling the substrates to the active site (Figure 4C). PET30 shows a less hydrophobic surrounding of the catalytic pocket when compared to the structures of Type I and Type II PET-degrading enzymes (Joo et al., 2018). PET27 contains a bulkier hydrophobic domain on one of the sides of the channel. This feature might influence accommodation of the substrate and activity on the polymers. A similar catalytic pocket was predicted for PET28 and PET29 (Supplementary Figure 3). Although active on BHET and PCL, these enzymes showed no measurable activity on PET. The enzymes derived from the human gut present a larger hydrophobic surface around the catalytic site, especially PET58 and PET59 (Supplementary Figure 3B).

### Bacteroidetal Polyethylene Terephthalate-Degrading Esterases Forming Two Phylogenetic Subclusters Are Globally Occurring Enzymes

**FIGURE 5:**
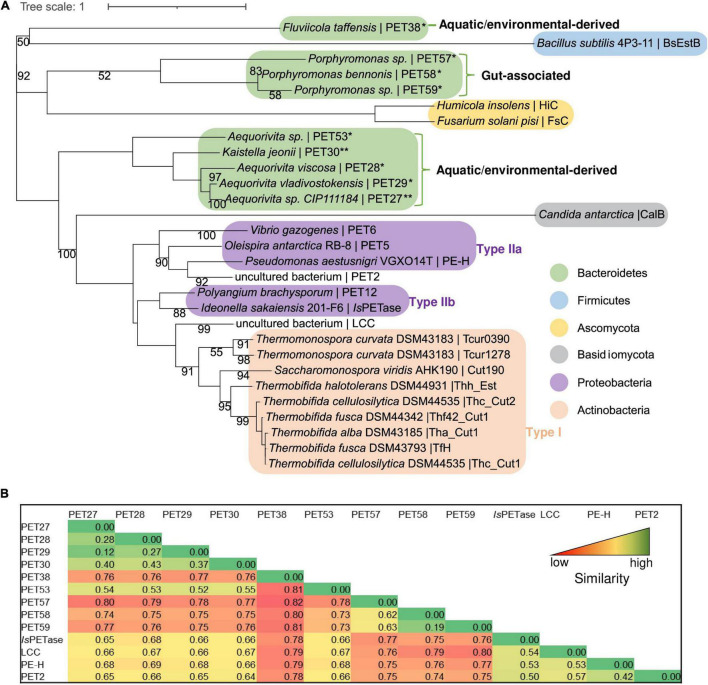
Phylogenetic tree and pairwise comparison of known PETases and bacteroidetal PET-hydrolyzing enzymes. **(A)** The tree was constructed using the RAxML-NG autoMRE algorithm (Kozlov et al., 2019) with the treesapp create command implemented in TreeSAPP (Morgan-Lang et al., 2020) with maximum bootstraps set at 1,000. GenBank entries of the putative and verified PET-active enzymes affiliated with the Bacteroidetes phylum are listed in Table 1; the entries of all other PETases included in the tree are indicated in Supplementary Table 3. The term “uncultured bacterium” refers to a not further specified microorganism derived from a metagenome or a mixed microbial consortium. **(B)** Heatmap representation of pairwise similarities between all enzymes affiliated with the Bacteroidetes phylum in comparison with four known PETases (*Is*PETase, LCC, PE-H, and PET2). The pairwise comparison was performed in MEGAX with the *p*-distance model. High values indicating low similarity are colored in red, low values indicating high similarity are colored in green.

Using the amino acid sequences of published and functionally verified PETases and employing the RAxML-NG autoMRE algorithm via TreeSAPP (Morgan-Lang et al., 2020), aphylogenetic analysis was performed. Multiple phylogenetic clusters formed that roughly corresponded to Actinobacteria, Proteobacteria, Firmicutes and Ascomycota (Figure 5A). While the putative and now confirmed Bacteroidetal PET-degrading hydrolases appear to be polyphyletic when added into the tree, distinct subclusters were formed. Notably, the two enzymes PET27 and PET30, shown to be active on PET foil, were grouped as part of subcluster that consisted of predicted and functional enzymes affiliated with genera *Aequorivita* and *Kaistella*. Furthermore, the predicted but functionally not verified enzymes from the genus *Porphyromonas* (PET57-PET59) formed a separate subcluster. Interestingly, these two subclusters harbored only sequences of aquatic and environmental origin orgut-affiliated sequences, respectively. Overall, sequences derived from Bacteroidetes seem to group by both taxonomy and environment, though low bootstrap values do not show a high degree of confidence. However, the interleaving of Bacteroidetal sequences from both a Firmicute, *B. subtilis*, and the eukaryotic Ascomycota sequences may suggest that PET-active enzymes are widely distributed phylogenetically, and further characterization studies resulting in additions to the tree are likely to provide better phylogenetic resolution. Additionally, the pairwise distance on the level computed in MEGAX with the p-distance model (Figure 5B) confirmed these groupings, with the highest similarity, as indicated by low pairwise distanced, occurring within the subclusters described. This analysis also indicated rather low similarity between the putative bacteroidetal PETases and the known PETases (*Is*PETase, LCC, PE-H, and PET2), mounting further evidence for the wide phylogenetic distribution of these enzymes.

The diversity of bacteroidetal enzymes acting on PET raised the question to what extent these enzymes could impact plastic degradation in the environment. To address this question in part, we analyzed the global distribution of PET27 and PET30 and their homologs. The protein sequences of PET27 and PET30 were analyzed for their occurrence and frequency in global databases available in IMG/M ER (Woyke et al., 2011; Mukherjee et al., 2020). Using both enzymes for a BLASTp-based search (cutoffs 50% identity; 80% coverage), we were initially able to identify very few (<10) possible homologs in the global databases analyzed and affiliated with the genera *Aequorivita* and *Kaistella* (Figure 1A). Interestingly, when we extended our search to the Flavobacterium-Cytophaga-Bacteroidetes (FCB), we were able to identify 98 possible homologs in our global searches including single-cell amplified genomes (SAGs) from the Baltic Sea (Supplementary Table 5). 47 hits were affiliated with the genus of the *Marinimicrobia* (candidate phylum of the FCB group), indicating a potential role for these ubiquitous and abundant marine microorganisms in PET-degradation. Others were more closely associated with Bacteroidetes (Austin et al., 2018). As expected, the majority of these homologs were associated with marine and aquatic samples (Figure 1B).

### *Kaistella jeonii* Is Able to Colonize Polyethylene Terephthalate Surfaces

These metagenomic analyses showed that *K. jeonii*, the organism from which PET30 originates, exists primarily in aquatic habitats. To illustrate that it not only exists there, but is also able to actively colonize PET surfaces, a culture of this organism was incubated with PET foil platelets. The foil sections were incubated for up to 1 week in R2A medium with *K. jeonii*, removed from the culture, and washed three times with buffer before staining with LIVE/DEAD staining solution. Samples were investigated under the confocal laser scanning microscope Axio Observer Z1/7, LSM 800 by Carl Zeiss Microscopy GmbH (Jena, Germany). Cells visible in Supplementary Figure 5 resisted repeated washing, indicating that they adhere under these laboratory conditions to the surface and an increasing number of cells was visible on the amorphous PET-foil after 5–7 days. The exact mechanism of attachment is not known. Nonetheless, it can be speculated that SusD binding modules, which are crucial for sugar polymer binding, could be involved. They can be found in both genomes of *K. jeonii* (GenBank acc. no. WP_039349586.1; RagB/SusD family nutrient uptake outer membrane protein) and *Aequorivita* sp. CIP111184 (GenBank acc. no. WP_111879847.1; SusD/RagB family nutrient-binding outer membrane lipoprotein).

## Discussion

Currently, only a handful of known bacterial phyla are known to produce active PET-esterases (Figure 5 and Supplementary Table 3). Here, we have identified and partially characterized two novel functional PET-hydrolyzing enzymes affiliated with the *Kaistella* and *Aequorivita* genera within the Bacteroidetes phylum. Bacteria belonging to the genus *Kaistella* are globally occurring aerobic organisms colonizing a wide range of different habitats including plants, soil, fish, the human gut, and sea water. Within the genus *Kaistella*, over one hundred species have been described of which few are pathogens, but many are beneficial and host-associated (Bernardet et al., 2005; Loch and Faisal, 2015). Only a few species have been identified within the genus *Aequorivita*, mainly belonging to marine or fresh-water organisms that are mostly psychrotolerant and aerobic (Bowman and Nichols, 2002). Notably, Bacteroidetes have been described as very potent degraders of polymers, and they harbor a multitude of hydrolases and binding modules (Dodd et al., 2011; Thomas et al., 2011; Foley et al., 2016).

The enzymes PET27 and PET30 characterized here are both typical esterases (i.e., serine hydrolases) belonging to the EC 3.1. Both appear to be secreted enzymes as they carry an C-terminal secretion signal linked to the transport into the periplasm (Desvaux et al., 2009) and one secretion PorC-like motif which is related to the type IX secretion system (T9SS) (Sato et al., 2010; de Diego et al., 2016). The T9SS is composed of several outer membrane, periplasmic and inner membrane proteins, whereby it is responsible for the secretion of pathogenicity factors, hydrolases and also for gliding motility in the Bacteroidetes phylum (Sato et al., 2010; de Diego et al., 2016).

PET27 and PET30 were active on PET foil, but differed strongly in their overall activities. PET27 contains a Phe-Met-Trp motif and PET30 a Phe-Met-Tyr. The most active enzymes such as LCC and *Is*PETase both carry a Tyr-Met-Trp consensus binding motif while the non-active enzymes PET38, PET53, PET57 PET58 and PET59 revealed either a Phe-Met-Ala, a Phe-Met-Trp or a Trp-Met-Tyr substrate binding motif. PET30 mutants, in which the amino acids were adjusted according to the active PETases, did not show the expected increase in activity. Therefore, we assume that not only individual amino acids are decisive, but rather that an interplay of hydrophobicity, location and accessibility of the catalytic triad is crucial for whether polymers can be degraded (Figure 4).

Benchmarking activities of polymer active enzymes with literature values is not trivial since most studies use different types of foils with different degrees of crystallinity and distinct assay conditions. To partially overcome this challenge, we produced our own recombinant wildtype enzymes of the *Is*PETase and compared its activities with PET27 and PET30. As expected, *Is*PETase was 4.7-fold more active at 30°C than PET27 and up to 253-fold more active than PET30. With respect to the overall activity of the *Is*PETase, however, our data are in line with published data for this enzyme (Son et al., 2019). The observation here that the activities of the PET27 and PET30 enzymes are relatively low compared to the *Is*PETase and certainly with respect to the published values of the even more active LCC imply that PET27 and PET30 are not PET esterases *in sensu strictu.* However, our data imply that both are short-chain fatty acid acting esterases revealing promiscuity in their substrate profile (Table 3 and Figure 3).

Intriguingly, the observation that both enzymes were catalytically active on PET foil could imply a wider role in the degradation of PET and especially PET nanoparticles. Because of the significant activities even at 4°C, these enzymes may in fact play a yet unknown role in long-term degradation of PET microparticles in cold environments. This hypothesis is supported by our observations that homologs of both enzymes can be found on a global level covering a wide range of climate zones (Figure 1) and that at least the cultivable *K. jeonii* is able to attach to PET surfaces.

In summary, our biochemical results significantly extend the knowledge of PET-degrading enzymes and provide promising candidates for biotechnological applications at low temperatures. Furthermore, the data presented here will help to advance our knowledge on the ecological role of the Bacteroidetes in the decomposition of marine PET litter and enable the development of an expanded phylogenetic framework for identifying the diversity of putative PETases in diverse marine microbial groups throughout the global ocean.

## Data Availability Statement

The original contributions presented in the study are included in the article/Supplementary Material, further inquiries can be directed to the corresponding author/s.

## Author Contributions

WS, JC, and PP-G designed the study, contributed to manuscript writing and designing bioassays. HZ and RD conducted experiments and contributed to planning and writing. PP-G, JC, and HZ were involved in enzyme structural work, bioinformatics, and initial phylogenetic analyses. VA, JS, and SSm conducted crystallization and structure determination. CS and JP contributed to planning and corrections. RS and CC were involved in global data base searches. DD performed HMM searches. SW and BH delivered clone producing the *Is*PETase. LP did microscopic analyses. AA was involved in gut microbiome data mining. SSt and SH were involved in phylogenetic analysis. All authors contributed to manuscript writing and editing.

## Conflict of Interest

SH is a co-founder of Koonkie Inc., a bioinformatics consulting company that designs and provides scalable algorithmic and data analytics solutions in the cloud. The remaining authors declare that the research was conducted in the absence of any commercial or financial relationships that could be construed as a potential conflict of interest.

## Publisher’s Note

All claims expressed in this article are solely those of the authors and do not necessarily represent those of their affiliated organizations, or those of the publisher, the editors and the reviewers. Any product that may be evaluated in this article, or claim that may be made by its manufacturer, is not guaranteed or endorsed by the publisher.

## Footnotes

1
https://www.ncbi.nlm.nih.gov/

2
http://www.uniprot.org/

3
http://jgi.doe.gov/

4
http://hmmer.org

5
https://geohack.toolforge.org

6
http://www.qgis.org

7
www.cgl.ucsf.edu/chimera

8
www.ebi.ac.uk/msd-srv/ssm/

## Supplementary Material

Supplementary Figure 1 Further biochemical characterization of PET27 and PET30 using *p*NP-substrates. Cofactor requirements were tested for PET27 with *p*NP-C8 at 40°C and for PET30 with *p*NP-C6 at 30°C **(A)**. For PET30, all other tests were carried out with *p*NP-C6 and all assays except **(B)** were conducted at 30°C. Thermostability of PET30 was assessed at 50 and 60°C over 3 h with *p*NP-C6 **(B)**. Inhibitors **(C)** and detergents **(D)** generally decrease the activity of PET30, particularly with higher concentrations. Activity of PET30 and PET30ΔPorC were compared under the same conditions using *p*NP-C6 **(E)**. Data represent mean values of at least three independent measurements.

Supplementary Figure 2 Zoom into the active site of PET30ΔPorC. Overlaid are the structures of PET30ΔPorC (blue) with the PETase from *I. sakaiensis* (light yellow, black labeling) in complex with HEMT (purple; PDB code 5XH3). The residues involved in binding are highlighted and numbered according to their structure. *The structure 5XH3 is a mutant where the catalytic Serine was mutated to an Alanine.

Supplementary Figure 3 Structure prediction models of verified and predicted PETases affiliated with the phylum of the Bacteroidetes. **(A)** 3D structures were modeled using the Robetta server using the *Is*PETase crystal structure (light yellow, 6EQE) as a backbone. For PET30, the crystal structure was shown. **(B)** Surface hydrophobicity around the tunnel leading to the active site of putative bacteroidetal PETases and functionally verified PET-degrading enzymes. Hydrophilic regions are displayed in turquoise and hydrophobic in gold.

Supplementary Figure 4 Amino acid alignment of 9 potential PETases affiliated with the Bacteroidetes phylum. The original sequences were used for the structural alignment and the alignment was constructed with T-Coffee. Alignment was visualized with Bioedit version 7.0.5. The *Is*PETase was included for reasons of benchmarking. Blue arrows indicate the start and the end of the active PET27 and PET30 clones. We introduced a methionine as the first aa of the protein sequences. The signal peptide deleted version of the enzymes was functionally verified. The red arrow labeled with CS indicates the predicted cleavage site of C-terminal PorC domain (Lasica et al., 2017). The gray arrow indicates the C-terminus of the truncated version of PET30 (PET30Δ300-366), number indicates the position of amino acid). PET30Δ300-366 was active on PCL and BHET.

Supplementary Figure 5 Confocal microscopic pictures of *K. jeonii* colonizing PET foil. The pictures were taken after 5–7 days of incubation of *K. jeonii* in R2A medium. Cells are dyed with LIVE/DEAD™ stain. Green fluorescence shows living cells, red fluorescence indicates dead cells. 2D front pictures were taken with Axio Observer Z1/7, LSM 800 (Carl Zeiss, Jena, Germany) of a 3D Z-stack image.

## References

[B1] Acero E. H. Ribitsch D. Steinkellner G. Gruber K. Greimel K. Eiteljoerg I. (2011). Enzymatic surface hydrolysis of PET: effect of structural diversity on kinetic properties of cutinases from *Thermobifida*. *Macromolecules* 44 4632–4640. 10.1021/ma200949p

[B2] Afonine P. V. Grosse-Kunstleve R. W. Echols N. Headd J. J. Moriarty N. W. Mustyakimov M. (2012). Towards automated crystallographic structure refinement with phenix. refine. *Acta Crystallogr. D Biol. Crystallogr.* 68 352–367. 10.1107/S0907444912001308 22505256 PMC3322595

[B3] Agarwala R. Barrett T. Beck J. Benson D. A. Bollin C. Bolton E. (2016). Database resources of the national center for biotechnology information. *Nucleic Acids Res.* 44 D7–D19. 26615191 10.1093/nar/gkv1290 PMC4702911

[B4] Almagro Armenteros J. J. Tsirigos K. D. Sønderby C. K. Petersen T. N. Winther O. Brunak S. (2019). SignalP 5.0 improves signal peptide predictions using deep neural networks. *Nat. Biotechnol.* 37 420–423. 10.1038/s41587-019-0036-z 30778233

[B5] Almeida A. Nayfach S. Boland M. Strozzi F. Beracochea M. Shi Z. J. (2021). A unified catalog of 204,938 reference genomes from the human gut microbiome. *Nat. Biotechnol.* 39 105–114. 10.1038/s41587-020-0603-3 32690973 PMC7801254

[B6] Austin H. P. Allen M. D. Donohoe B. S. Rorrer N. A. Kearns F. L. Silveira R. L. (2018). Characterization and engineering of a plastic-degrading aromatic polyesterase. *Proc. Natl. Acad. Sci. U.S.A.* 115 E4350–E4357. 10.1073/pnas.1718804115 29666242 PMC5948967

[B7] Berman H. M. Westbrook J. Feng Z. Gilliland G. Bhat T. N. Weissig H. I. (2000). The protein data bank. *Nucleic Acids Res.* 28 235–242. 10592235 10.1093/nar/28.1.235 PMC102472

[B8] Bernardet J. F. Vancanneyt M. Matte-Tailliez O. Grisez L. Tailliez P. Bizet C. (2005). Polyphasic study of *Chryseobacterium* strains isolated from diseased aquatic animals. *Syst. Appl. Microbiol.* 28 640–660. 10.1016/j.syapm.2005.03.016 16156122

[B9] Bollinger A. Thies S. Knieps-Grunhagen E. Gertzen C. Kobus S. Hoppner A. (2020). A novel polyester hydrolase from the marine bacterium *Pseudomonas aestusnigri*–structural and functional insights. *Front. Microbiol.* 11:114. 10.3389/fmicb.2020.00114 32117139 PMC7031157

[B10] Bowman J. P. Nichols D. S. (2002). *Aequorivita* gen. nov., a member of the family Flavobacteriaceae isolated from terrestrial and marine Antarctic habitats. *Int. J. Syst. Evol. Microbiol.* 52 1533–1541. 10.1099/00207713-52-5-1533 12361255

[B11] Chen C.-C. Han X. Li X. Jiang P. Niu D. Ma L. (2021). General features to enhance enzymatic activity of poly (ethylene terephthalate) hydrolysis. *Nat. Catal.* 4 425–430. 10.1038/s41929-021-00616-y

[B12] Chen S. Tong X. Woodard R. W. Du G. Wu J. Chen J. (2008). Identification and characterization of bacterial cutinase. *J. Biol. Chem.* 283 25854–25862. 10.1074/jbc.m800848200 18658138 PMC3258855

[B13] NCBI Resource Coordinators (2017). Database resources of the national center for biotechnology information. *Nucleic Acids Res.* 45 D12–D17. 27899561 10.1093/nar/gkw1071 PMC5210554

[B14] Danso D. Chow J. Streit W. R. (2019). Plastics: microbial degradation, environmental and biotechnological perspectives. *Appl. Environ. Microbiol.* 85 1–14. 10.1007/978-3-030-48973-1_1 PMC6752018 31324632

[B15] Danso D. Schmeisser C. Chow J. Zimmermann W. Wei R. Leggewie C. (2018). New insights into the function and global distribution of polyethylene terephthalate (PET)-degrading bacteria and enzymes in marine and terrestrial metagenomes. *Appl. Environ. Microbiol.* 84:e02773-17. 10.1128/AEM.02773-17 29427431 PMC5881046

[B16] de Diego I. Ksiazek M. Mizgalska D. Koneru L. Golik P. Szmigielski B. (2016). The outer-membrane export signal of *Porphyromonas gingivalis* type IX secretion system (T9SS) is a conserved C-terminal β-sandwich domain. *Sci. Rep.* 6:23123. 10.1038/srep23123 27005013 PMC4804311

[B17] DeLano W. L. (2002). Pymol: an open-source molecular graphics tool. *CCP4 Newslett. Protein Crystallogr.* 40 82–92.

[B18] Desvaux M. Hébraud M. Talon R. Henderson I. R. (2009). Outer membrane translocation: numerical protein secretion nomenclature in question in mycobacteria. *Trends Microbiol.* 17 338–340. 10.1016/j.tim.2009.05.008 19674902

[B19] Dodd D. Mackie R. I. Cann I. K. O. (2011). Xylan degradation, a metabolic property shared by rumen and human colonic Bacteroidetes. *Mol. Microbiol.* 79 292–304. 10.1111/j.1365-2958.2010.07473.x 21219452 PMC4561535

[B20] Emsley P. Cowtan K. (2004). Coot: model-building tools for molecular graphics. *Acta Crystallogr. D Biol. Crystallogr.* 60 2126–2132. 10.1107/s0907444904019158 15572765

[B21] Foley M. H. Cockburn D. W. Koropatkin N. M. (2016). The Sus operon: a model system for starch uptake by the human gut Bacteroidetes. *Cell. Mol. Life Sci.* 73 2603–2617. 10.1007/s00018-016-2242-x 27137179 PMC4924478

[B22] Geyer R. Jambeck J. R. Law K. L. (2017). Production, use, and fate of all plastics ever made. *Sci. Adv.* 3:e1700782. 10.1126/sciadv.1700782 28776036 PMC5517107

[B23] Haernvall K. Zitzenbacher S. Amer H. Zumstein M. T. Sander M. McNeill K. (2017). Polyol structure influences enzymatic hydrolysis of bio-based 2,5-furandicarboxylic acid (FDCA) polyesters. *Biotechnol. J.* 12:1600741. 10.1002/biot.201600741 28731613

[B24] Hahnke R. L. Meier-Kolthoff J. P. García-López M. Mukherjee S. Huntemann M. Ivanova N. N. (2016). Genome-based taxonomic classification of bacteroidetes. *Front. Microbiol.* 7:2003. 10.3389/fmicb.2016.02003 28066339 PMC5167729

[B25] Hall T. A. (1999). *BioEdit: A User-Friendly Biological Sequence Alignment Editor and Analysis Program for Windows 95/98/NT. Nucleic Acids Symposium Series.* London: Information Retrieval Ltd., c1979–c2000.

[B26] Han X. Liu W. Huang J. W. Ma J. Zheng Y. Ko T. P. (2017). Structural insight into catalytic mechanism of PET hydrolase. *Nat. Commun.* 8:2106. 10.1038/s41467-017-02255-z PMC5727383 29235460

[B27] Hu X. Thumarat U. Zhang X. Tang M. Kawai F. (2010). Diversity of polyester-degrading bacteria in compost and molecular analysis of a thermoactive esterase from *Thermobifida alba* AHK119. *Appl. Microbiol. Biotechnol.* 87 771–779. 10.1007/s00253-010-2555-x 20393707

[B28] Jambeck J. R. Geyer R. Wilcox C. Siegler T. R. Perryman M. Andrady A. (2015). Plastic waste inputs from land into the ocean. *Science* 347 768–771. 10.1126/science.1260352 25678662

[B29] Joo S. Cho I. J. Seo H. Son H. F. Sagong H. Y. Shin T. J. (2018). Structural insight into molecular mechanism of poly(ethylene terephthalate) degradation. *Nat. Commun.* 9:382. 10.1038/s41467-018-02881-1 PMC5785972 29374183

[B30] Kabsch W. (2014). Processing of X-ray snapshots from crystals in random orientations. *Acta Crystallogr. D Biol. Crystallogr.* 70 2204–2216. 10.1107/S1399004714013534 25084339 PMC4118830

[B31] Kim D. E. Chivian D. Baker D. (2004). Protein structure prediction and analysis using the Robetta server. *Nucleic Acids Res.* 32 W526–W531. 15215442 10.1093/nar/gkh468 PMC441606

[B32] Kleeberg I. Hetz C. Kroppenstedt R. M. Muller R. J. Deckwer W. D. (1998). Biodegradation of aliphatic-aromatic copolyesters by *Thermomonospora fusca* and other thermophilic compost isolates. *Appl. Environ. Microbiol.* 64 1731–1735. 10.1128/AEM.64.5.1731-1735.1998 9572944 PMC106223

[B33] Kozlov A. M. Darriba D. Flouri T. Morel B. Stamatakis A. (2019). RAxML-NG: a fast, scalable and user-friendly tool for maximum likelihood phylogenetic inference. *Bioinformatics* 35 4453–4455. 10.1093/bioinformatics/btz305 31070718 PMC6821337

[B34] Krieg N. Ludwig W. Euzéby J. Whitman W. (2015). “Bacteroidetes phyl. nov,” in *Bergey’s Manual of Systematics of Archaea and Bacteria*, eds Trujillo M. E. Dedysh S. DeVos P. (Hoboken, NJ: John Wiley & Sons, Inc.), 1–2. 10.1002/9781118960608.pbm00004

[B35] Lasica A. M. Ksiazek M. Madej M. Potempa J. (2017). The type IX secretion system (T9SS): highlights and recent insights into its structure and function. *Front. Cell. Infect. Microbiol.* 7:215. 10.3389/fcimb.2017.00215 28603700 PMC5445135

[B36] Letunic I. Bork P. (2019). Interactive tree of life (iTOL) v4: recent updates and new developments. *Nucleic Acids Res.* 47 W256–W259. 10.1093/nar/gkz239 30931475 PMC6602468

[B37] Li N. Zhu Y. LaFrentz B. R. Evenhuis J. P. Hunnicutt D. W. Conrad R. A. (2017). The type IX secretion system is required for virulence of the fish pathogen *Flavobacterium columnare*. *Appl. Environ. Microbiol.* 83:e01769-17. 10.1128/AEM.01769-17 PMC5691404 28939608

[B38] Liebschner D. Afonine P. V. Baker M. L. Bunkóczi G. Chen V. B. Croll T. I. (2019). Macromolecular structure determination using X-rays, neutrons and electrons: recent developments in Phenix. *Acta Crystallogr. D Struct. Biol.* 75 861–877. 10.1107/S2059798319011471 31588918 PMC6778852

[B39] Loch T. P. Faisal M. (2015). Emerging flavobacterial infections in fish: a review. *J. Adv. Res.* 6 283–300. 10.1016/j.jare.2014.10.009 26257926 PMC4522593

[B40] Markowitz V. M. Chen I. M. Palaniappan K. Chu K. Szeto E. Grechkin Y. (2012). IMG: the integrated microbial genomes database and comparative analysis system. *Nucleic Acids Res.* 40 D115–D122. 22194640 10.1093/nar/gkr1044 PMC3245086

[B41] Matlab S. (2012). *Matlab.* Natick, MA: The MathWorks.

[B42] Mistry J. Finn R. D. Eddy S. R. Bateman A. Punta M. (2013). Challenges in homology search: HMMER3 and convergent evolution of coiled-coil regions. *Nucleic Acids Res.* 41:e121. 10.1093/nar/gkt263 23598997 PMC3695513

[B43] Mitchell A. L. Almeida A. Beracochea M. Boland M. Burgin J. Cochrane G. (2019). MGnify: the microbiome analysis resource in 2020. *Nucleic Acids Res.* 48 D570–D578. 10.1093/nar/gkz1035 31696235 PMC7145632

[B44] Miyakawa T. Mizushima H. Ohtsuka J. Oda M. Kawai F. Tanokura M. (2015). Structural basis for the Ca ^2+^-enhanced thermostability and activity of PET-degrading cutinase-like enzyme from *Saccharomonospora viridis* AHK190. *Appl. Microbiol. Biotechnol.* 99 4297–4307. 10.1007/s00253-014-6272-8 25492421

[B45] Molitor R. Bollinger A. Kubicki S. Loeschcke A. Jaeger K. E. Thies S. (2020). Agar plate-based screening methods for the identification of polyester hydrolysis by *Pseudomonas* species. *Microb. Biotechnol.* 13 274–284. 10.1111/1751-7915.13418 31016871 PMC6922526

[B46] Morgan-Lang C. McLaughlin R. Armstrong Z. Zhang G. Chan K. Hallam S. J. (2020). TreeSAPP: the tree-based sensitive and accurate phylogenetic profiler. *Bioinformatics* 36 4706–4713. 10.1093/bioinformatics/btaa588 32637989 PMC7695126

[B47] Mukherjee S. Stamatis D. Bertsch J. Ovchinnikova G. Sundaramurthi J. C. Lee J. (2020). Genomes OnLine Database (GOLD) v.8: overview and updates. *Nucleic Acids Res.* 49 D723–D733. 10.1093/nar/gkaa983 33152092 PMC7778979

[B48] Mulnaes D. Porta N. Clemens R. Apanasenko I. Reiners J. Gremer L. (2020). TopModel: template-based protein structure prediction at low sequence identity using top-down consensus and deep neural networks. *J. Chem. Theory Comput.* 16 1953–1967. 10.1021/acs.jctc.9b00825 31967823

[B49] Munoz R. Rosselló-Móra R. Amann R. (2016). Revised phylogeny of Bacteroidetes and proposal of sixteen new taxa and two new combinations including Rhodothermaeota phyl. nov. *Syst. Appl. Microbiol.* 39 281–296. 10.1016/j.syapm.2016.04.004 27287844

[B50] Notredame C. Higgins D. G. Heringa J. (2000). T-Coffee: a novel method for fast and accurate multiple sequence alignment. *J. Mol. Biol.* 302 205–217. 10964570 10.1006/jmbi.2000.4042

[B51] Numoto N. Kamiya N. Bekker G.-J. Yamagami Y. Inaba S. Ishii K. (2018). Structural dynamics of the PET-degrading cutinase-like enzyme from *Saccharomonospora viridis* AHK190 in substrate-bound states elucidates the Ca^2+^-driven catalytic cycle. *Biochemistry* 57 5289–5300. 10.1021/acs.biochem.8b00624 30110540

[B52] Ollis D. L. Cheah E. Cygler M. Dijkstra B. Frolow F. Franken S. M. (1992). The alpha/beta hydrolase fold. *Protein Eng.* 5 197–211. 1409539 10.1093/protein/5.3.197

[B53] Pérez-García P. Danso D. Zhang H. Chow J. Streit W. R. (2021). Exploring the global metagenome for plastic-degrading enzymes. *Methods Enzymol.* 648 137–157. 10.1016/bs.mie.2020.12.022 33579401

[B54] Ribitsch D. Herrero Acero E. Greimel K. Dellacher A. Zitzenbacher S. Marold A. (2012). A new esterase from *Thermobifida halotolerans* hydrolyses polyethylene terephthalate (PET) and polylactic acid (PLA). *Polymers* 4 617–629. 10.3390/polym4010617

[B55] Ronkvist ÅM. Xie W. Lu W. Gross R. A. (2009). Cutinase-catalyzed hydrolysis of poly(ethylene terephthalate). *Macromolecules* 42 5128–5138.

[B56] Sato K. Naito M. Yukitake H. Hirakawa H. Shoji M. McBride M. J. (2010). A protein secretion system linked to bacteroidete gliding motility and pathogenesis. *Proc. Natl. Acad. Sci. U.S.A.* 107 276–281. 10.1073/pnas.0912010107 19966289 PMC2806738

[B57] Shoji M. Sato K. Yukitake H. Kondo Y. Narita Y. Kadowaki T. (2011). Por secretion system-dependent secretion and glycosylation of *Porphyromonas gingivalis* hemin-binding protein 35. *PLoS One* 6:e21372. 10.1371/journal.pone.0021372 21731719 PMC3120885

[B58] Son H. F. Cho I. J. Joo S. Seo H. Sagong H.-Y. Choi S. Y. (2019). Rational protein engineering of thermo-stable PETase from *Ideonella sakaiensis* for highly efficient PET degradation. *ACS Catal.* 9 3519–3526.

[B59] Studier F. W. (2005). Protein production by auto-induction in high-density shaking cultures. *Protein Exp. Purif.* 41 207–234. 10.1016/j.pep.2005.01.016 15915565

[B60] Sulaiman S. Yamato S. Kanaya E. Kim J. J. Koga Y. Takano K. (2012). Isolation of a novel cutinase homolog with polyethylene terephthalate-degrading activity from leaf-branch compost by using a metagenomic approach. *Appl. Environ. Microbiol.* 78 1556–1562. 10.1128/AEM.06725-11 22194294 PMC3294458

[B61] Sulaiman S. You D. J. Kanaya E. Koga Y. Kanaya S. (2014). Crystal structure and thermodynamic and kinetic stability of metagenome-derived LC-cutinase. *Biochemistry* 53 1858–1869. 10.1021/bi401561p 24593046

[B62] The UniProt Consortium (2017). UniProt: the universal protein knowledgebase. *Nucleic Acids Res.* 45 D158–D169. 27899622 10.1093/nar/gkw1099 PMC5210571

[B63] Thomas F. Hehemann J. H. Rebuffet E. Czjzek M. Michel G. (2011). Environmental and gut Bacteroidetes: the food connection. *Front. Microbiol.* 2:16. 10.3389/fmicb.2011.00093 21747801 PMC3129010

[B64] Wei R. Oeser T. Zimmermann W. (2014). Synthetic polyester-hydrolyzing enzymes from thermophilic actinomycetes. *Adv. Appl. Microbiol.* 89 267–305. 10.1016/B978-0-12-800259-9.00007-X 25131405

[B65] Wei R. Zimmermann W. (2017). Microbial enzymes for the recycling of recalcitrant petroleum-based plastics: how far are we? *Microb. Biotechnol.* 10 1308–1322. 10.1111/1751-7915.12710 28371373 PMC5658625

[B66] Wexler H. M. (2007). *Bacteroides*: the good, the bad, and the nitty-gritty. *Clin. Microbiol. Rev.* 20 593–621. 10.1128/CMR.00008-07 17934076 PMC2176045

[B67] Woyke T. Chertkov O. Lapidus A. Nolan M. Lucas S. Del Rio T. G. (2011). Complete genome sequence of the gliding freshwater bacterium *Fluviicola taffensis* type strain (RW262). *Stand. Genomic Sci.* 5 21–29. 10.4056/sigs.2124912 22180807 PMC3236050

[B68] Wright R. J. Bosch R. Langille M. G. Gibson M. I. Christie-Oleza J. A. (2021). A multi-OMIC characterisation of biodegradation and microbial community succession within the PET plastisphere. *Microbiome* 9 1–22. 10.1155/2021/6620574 34154652 PMC8215760

[B69] Yoshida S. Hiraga K. Takehana T. Taniguchi I. Yamaji H. Maeda Y. (2016). A bacterium that degrades and assimilates poly(ethylene terephthalate). *Science* 351 1196–1199. 10.1126/science.aad6359 26965627

