# Supplementary material for: The Bacteroidetes Aequorivita sp. and Kaistella jeonii Produce Promiscuous Esterases With PET-Hydrolyzing Activity
Source: Front Microbiol. 2022 Jan 5;12:803896. doi: 10.3389/fmicb.2021.803896 (PMC8767016; doi:10.3389/fmicb.2021.803896)
Supplement: Supplementary file 1 [file Data_Sheet_1.PDF]

TABLE S1: Bacterial strains and plasmids used in this work.

Strain	Properties	Reference/source
E. coli DH5α	*supE*44 Δ*lacU*169 (Φ80 lac*Z* ΔM15) *hsdR*17 *recA*1 *endA*1 *gyrA*96 *thi*-1 *relA*1	Invitrogen (Karlsruhe, Germany)
E. coli BL21 (DE3)	F^-, *ompT*, *hsdS* B (r_B^- m_B^-) *gal*, *dcm*, λDE3	Novagen/Merck (Darmstadt, Germany)
E. coli SHuffle® T7	*huA*2 *lacZ*::T7 gene1 [lon] *ompT ahpC gal* λatt::pNEB3-r1-cDsbC (SpecR, lacIq) Δ*trxB sulA*11 R(mcr-73::miniTn10--TetS)2 [dcm] R(zgb-210::Tn10 --TetS) *endA*1 Δ*gor* Δ(*mcrC-mrr*)114::IS10	NEB (Frankfurt am Main, Germany)

Vector	Properties	Reference/source
pET21a(+)	Expression vector, *lacI*, Amp^R, T7-*lac*- promoter, C-terminal His_6-tag coding sequence	Novagen/Merck (Darmstadt, Germany)
pET21a(+)::PET27	1026 bp insert in pET21a(+) coding for PET27	This work
pET21a(+)::PET28	1029 bp insert in pET21a(+) coding for PET28	This work
pET21a(+)::PET29	1029 bp insert in pET21a(+) coding for PET29	This work
pET21a(+)::PET30	1032 bp insert in pET21a(+) coding for PET30	This work
pET21a(+)::PET30_ Δ300-366	831 bp insert in pET21a(+) coding for PET30, truncated protein lacking PorC	This work
pET21a(+)::PET38	1341 bp insert in pET21a(+) coding for PET38	This work
pET21a(+)::PET53	1072 bp insert in pET21a(+) coding for PET53	This work
pET21a(+)::PET57	969 bp insert in pET21a(+) coding for PET57	This work
pET21a(+)::PET58	1014 bp insert in pET21a(+) coding for PET58	This work
pET21a(+)::PET59	1035 bp insert in pET21a(+) coding for PET59	This work
pMAL-p4x::IsPETase	795 bp insert in pMAL-p4x coding for the wildtype *Is*PETase from *Ideonella sakaiensis*	This work

TABLE S2: Primers used in this work.

Primer	Sequence (5' → 3')	Length (bp)	Tm (°C)	Source
T7_prom	TAATACGACTCACTATAGGG	20	53	Eurofins MWG (Elsberg, Germany)
T7_term	CTAGTTATTGCTCAGCGGT	19	54	Eurofins MWG (Elsberg, Germany)
PET_for	ATATAGGCGCCAGCAACC	18	59	Novagen/Merck (Darmstadt, Germany)
PET_rev	TCCGGATATAGTTCCTC	17	52	Novagen/Merck (Darmstadt, Germany)

TABLE S3: Identifiers and GenBank entries employed in the phylogenetic clustering of PET active enzymes in FIGURE 4.

Name	GenBank or PDB entry	Phylogenetic Affiliation	Reference
BsEstB	ADH43200.1	*Bacillus subtilis* 4P3-11	Herrero Acero *et al*. 2011
CalB	4K6G_A	*Candida antarctica*	Andersen *et al*. 1999; Xie *et al*. 2014
Cut190	BAO42836.1	*Saccharomonospora viridis* AHK190	Kawai *et al*. 2014
FsC	1CEX	*Fusarium solani* pisi	Silva *et al*. 2005
HiC	4OYY	*Humicola insolens*	Ronkvist *et al*. 2009
*Is*PETase	GAP38373.1	*Ideonella sakaiensis* 201-F6	Yoshida *et al*. 2016
LCC	AEV21261.1	uncultured bacterial species (leaf-branch compost)	Sulaiman *et al*. 2012
PE-H	A0A1H6AD45	*Pseudomonas aestusnigri* VGXO14T	Bollinger *et al*. 2020
PET2	C3RYL0	uncultured bacterium	
PET5	R4YKL9	*Oleispira antarctica* RB-8	Danso *et al*. 2018
PET6	A0A1Z2SIQ1	*Vibrio gazogenes*	
PET12	A0A0G3BI90	*Polyangium brachysporum*	
Tcur0390	WP_012850775.1	*Thermomonospora curvata* DSM43183	Wei *et al*. 2014
Tcur1278	WP_012851645.1	*Thermomonospora curvata* DSM43183	Chertkov *et al*. 2011
TfH	WP_011291330.1	*Thermobifida fusca* DSM43793	Müller *et al*. 2005
Thc_Cut1	ADV92526.1	*Thermobifida cellulosilytica* DSM44535	
Thc_Cut2	ADV92527.1	*Thermobifida cellulosilytica* DSM44535	Herrero Acero *et al*. 2011
Thf42_Cut1	ADV92528.1	*Thermobifida fusca* DSM44342	
Tha_Cut1	ADV92525.1	*Thermobifida alba* DSM43185	Ribitsch *et al*. 2012
Thh_Est	AFA45122.1	*Thermobifida halotolerans* DSM44931	

TABLE S4: Data collection and refinement statistics for PET30

	PET30
**Wavelength**	0.9795
**Resolution range**	38.8 - 2.1 (2.175 - 2.1)
**Space group**	P 43 21 2
**Unit cell**	109.755 109.755 41.803 90 90 90
**Total reflections**	267429 (24691)
**Unique reflections**	15447 (1505)
**Multiplicity**	17.3 (16.4)
**Completeness (%)**	99.95 (100.00)
**Mean I/sigma(I)**	13.95 (5.38)
**Wilson B-factor**	24.09
**R-merge**	0.1746 (0.7031)
**R-meas**	0.1799 (0.7255)
**R-pim**	0.04277 (0.1772)
**CC1/2**	0.997 (0.945)
**CC***	0.999 (0.986)
**Reflections used in refinement**	15443 (1507)
**Reflections used for R-free**	716 (78)
**R-work**	0.1619 (0.1655)
**R-free**	0.2187 (0.2460)
**CC(work)**	0.962 (0.901)
**CC(free)**	0.932 (0.840)
**Number of non-hydrogen atoms**	2208
**macromolecules**	2006
**solvent**	202
**Protein residues**	273
**RMS(bonds)**	0.011
**RMS(angles)**	1.21
**Ramachandran favored (%)**	95.57
**Ramachandran allowed (%)**	3.69
**Ramachandran outliers (%)**	0.74
**Rotamer outliers (%)**	0.00
**Clashscore**	5.01
**Average B-factor**	27.12
**macromolecules**	26.45
**solvent**	33.82

Statistics for the highest-resolution shell are shown in parentheses.

TABLE S5: Homologs of bacteroidetal PET27 and PET30 hydrolases in metagenomes.

Locus Tag	Gene ID	Genome Name (GREEN=Bacteroidetes; YELLOW=FCB)	NCBI Biosample Accession	Pubmed ID /GenBank entry
Ga0266410_112824	2790477168	*Aequorivita sp.* CPC67	SAMEA2621812	25999513
Ga0267287_12740	2786516536	*Marinimicrobia bacterium* SP4388	SAMEA2621812	25999513
Ga0214074_111348	2758370761	*Psychroflexus torquis* ATCC 700755	SAMN02603919	24391155 , 32431677
Ga0441744_095_43538_44635	2893010289	*Psychroflexus gondwanensis* ACAM 365	SAMN10790446	24391155, 32431677
Ga0452869_05_68007_69104	2890419200	*Brumimicrobium glaciale* IC156	SAMN10779751	24391155, 32431677
Ga0125537_104199	2776033716	*Altibacter lentus* JL2010	SAMN02988278	25342673
Ga0101260_1142	2663974789	*Bacteroidetes bacterium* SCGC AD-308-D03 - v2	SAMN06314724	373136
Ga0266415_110121	2790487960	*Aequorivita sp.* SAT106	SAMEA2620929	25999513
Ga0266677_12858	2778555477	*Flavobacteriaceae bacterium* SAT1509	SAMEA2620929	25999513
Ga0267170_100812	2814895288	*Gemmatimonadetes bacterium* RS373	SAMEA2620929	25999513
Ga0267250_100918	2786410539	*Marinimicrobia bacterium* RS418	SAMEA2620929	25999513
Ga0267252_10536	2786413511	*Marinimicrobia bacterium* RS816	SAMEA2620929	25999513
Ga0267252_10542	2786413517	*Marinimicrobia bacterium* RS816	SAMEA2620929	25999513
Ga0266410_112825	2769034576	*Aequorivita sp.* CPC68	SAMEA2621812	25999513
Ga0267287_12741	2767134878	*Marinimicrobia bacterium* SP4389	SAMEA2621812	25999513
Ga0214074_111349	2765235181	*Psychroflexus torquis* ATCC 700756	SAMN02603919	24391155 , 32431678
Ga0441744_095_43538_44636	2763335483	*Psychroflexus gondwanensis* ACAM 366	SAMN10790446	24391155, 32431677
Ga0452869_05_68007_69105	2761435786	*Brumimicrobium glaciale* IC157	SAMN10779751	24391155, 32431677
Ga0267200_1431	2786528832	*Marinimicrobia bacterium* EAC25	SAMEA2620855	25999513, 29337314
Ga0267200_1544	2786528992	*Marinimicrobia bacterium* EAC25	SAMEA2620855	25999513, 29337314
Ga0416765_13_4825_5925	2860314059	*Chryseobacterium sp.* 16F	SAMN14915977	25824943
Pl23P_05397	639004873	*Polaribacter irgensii* 23-P	SAMN02436114	9542092 /NZ_CH724148.1
Aeqsu_2514	2509583556	*Aequorivita sublithincola* QSSC9-3, DSM 14238	SAMN02232006	28604660
Ga0266584_11073	2788314349	*Cryomorphaceae bacterium* CPC63	SAMEA2622923	25999513
Ga0266589_10312	2787698016	*Cytophagia bacterium* NAT375	SAMEA2622923	25999513
Ga0267262_14611	2786499724	*Marinimicrobia bacterium* SAT24	SAMEA2622923	25999513
Ga0267263_11113	2786500508	*Marinimicrobia bacterium* SAT2619	SAMEA2622923	25999513
Ga0267267_12326	2786498443	*Marinimicrobia bacterium* SP108	SAMEA2622923	25999513
Ga0267268_10328	2786441642	*Marinimicrobia bacterium* SP173	SAMEA2622923	25999513
Ga0267268_1112	2786442127	*Marinimicrobia bacterium* SP173	SAMEA2622923	25999513
Ga0267270_13120	2786484524	*Marinimicrobia bacterium* SP276	SAMEA2622923	25999513
Ga0267272_1173	2786478779	*Marinimicrobia bacterium* SP3060	SAMEA2622923	25999513
Ga0267273_13417	2786477564	*Marinimicrobia bacterium* SP3097	SAMEA2622923	25999513
Ga0267276_13625	2786480397	*Marinimicrobia bacterium* SP3117	SAMEA2622923	25999513
Ga0267279_10440	2786489803	*Marinimicrobia bacterium* SP328	SAMEA2622923	25999513
Ga0267280_12433	2786520859	*Marinimicrobia bacterium* SP359	SAMEA2622923	25999513
Ga0267282_10178	2786519018	*Marinimicrobia bacterium* SP4039	SAMEA2622923	25999513
Ga0063505_10623	2606052547	*Roseivirga seohaensis aquiponti* D-25	SAMN03145748	27107724/ JSVA01000000

Ga0267161_105313	2814952585	*Gemmatimonadetes bacterium* EAC635	SAMEA2623295	25999513
Ga0267164_1577	2814955360	*Gemmatimonadetes bacterium* NAT196	SAMEA2623295	25999513
Ga0267234_12226	2786460090	*Marinimicrobia bacterium* NAT495	SAMEA2623295	25999513
Ga0267237_1518	2786446587	*Marinimicrobia bacterium* NAT62	SAMEA2623295	25999513
Ga0267238_1416	2786464404	*Marinimicrobia bacterium* NAT74	SAMEA2623295	25999513
Ga0267239_13724	2786462435	*Marinimicrobia bacterium* NP104	SAMEA2623295	25999513
Ga0215720_101116	2756777030	*Marinirhabdus gelatinilytica* DSM 101478	SAMN08776299	QRAO00000000
Ga0266586_11614	2788308996	*Cryomorphaceae bacterium* SP53	SAMEA2619927	25999513
Ga0267165_10476	2814943193	*Gemmatimonadetes bacterium* NP105	SAMEA2619818	25999513
SCB49_04680	641143976	*Ulvibacter sp.* SCB49	SAMN02981237	ABCO00000000
G440DRAFT_00359	2524126300	*Aequorivita capsosiphonis* DSM 23843	SAMN02440880	AUBG00000000
Ga0266370_16423	2789796087	*Altibacter sp.* EAC109	SAMEA2619376	25999513
Ga0266560_154	2825980380	*Crocinitomicaceae bacterium* NAT165	SAMEA2619376	25999513
Ga0267217_1484	2786425266	*Marinimicrobia bacterium* MED806	SAMEA2619376	25999513
Ga0267217_16831	2786425767	*Marinimicrobia bacterium* MED806	SAMEA2619376	25999513
Ga0267218_1219	2786426885	*Marinimicrobia bacterium* MED808	SAMEA2619376	25999513
Ga0267219_10943	2786427199	*Marinimicrobia bacterium* MED812	SAMEA2619376	25999513
Ga0267220_10096	2786428744	*Marinimicrobia bacterium* MED829	SAMEA2619376	25999513
Ga0267220_10106	2786428754	*Marinimicrobia bacterium* MED829	SAMEA2619376	25999513
Ga0267224_14310	2786401069	*Marinimicrobia bacterium* NAT217	SAMEA2619376	25999513
Ga0267226_12918	2786404156	*Marinimicrobia bacterium* NAT220	SAMEA2619376	25999513
Ga0267227_13041	2786403315	*Marinimicrobia bacterium* NAT224	SAMEA2619376	25999513
Ga0267228_1422	2786399119	*Marinimicrobia bacterium* NAT230	SAMEA2619376	25999513
Ga0267208_10372	2786507568	*Marinimicrobia bacterium* MED586	SAMEA2619667	25999513
Ga0267208_10412	2786507636	*Marinimicrobia bacterium* MED586	SAMEA2619667	25999513
Ga0267209_1018	2786508441	*Marinimicrobia bacterium* MED589	SAMEA2619667	25999513
Ga0267212_106014	2786417419	*Marinimicrobia bacterium* MED648	SAMEA2619667	25999513
Ga0267212_10712	2786417608	*Marinimicrobia bacterium* MED648	SAMEA2619667	25999513
Ga0267212_110812	2786418228	*Marinimicrobia bacterium* MED648	SAMEA2619667	25999513
Ga0267213_15817	2786422578	*Marinimicrobia bacterium* MED757	SAMEA2619667	25999513
Ga0267214_1214	2786420423	*Marinimicrobia bacterium* MED764	SAMEA2619667	25999513
Ga0267214_12210	2786420472	*Marinimicrobia bacterium* MED764	SAMEA2619667	25999513
Ga0267214_1225	2786420467	*Marinimicrobia bacterium* MED764	SAMEA2619667	25999513
Ga0267214_14025	2786421083	*Marinimicrobia bacterium* MED764	SAMEA2619667	25999513
Ga0344934_2405	2839770976	*Dokdonia sinensis* SH27	SAMN10250232	32228747
Ga0441973_01_775875_776969	2890600833	*Ulvibacter sp.* KK4	SAMD00166796	32539909
LY87DRAFT_1302	2597312806	*Dokdonia sp.* Hel_I_5	SAMN05661066	32539909
Ga0267202_1226	2786524372	*Marinimicrobia bacterium* EAC649	SAMEA2620230	25999513
Ga0267202_13210	2786524561	*Marinimicrobia bacterium* EAC649	SAMEA2620230	25999513
Ga0267202_13216	2786524567	*Marinimicrobia bacterium* EAC649	SAMEA2620230	25999513
Ga0350409_3222	2848310855	*Psychroflexus sp.* MES1-P1E	SAMN08125772	PJBS00000000, CM009131
Ga0066802_10753	2623289129	*Aequorivita viscosa* DSM 26349	SAMN04487908	FQYV00000000
Ga0077144_1092	2641186029	*Chryseobacterium jeonii* DSM 17048	SAMN03145167	JSYL00000000
Ga0079842_10854	2668214597	*Aequorivita viscosa* CGMCC 1.11023	SAMN05216556	FNNS00000000

Ga0104531_1111	2676887423	*Chryseobacterium jeonii* DSM 17048	SAMN05421876	FOLA00000000
Ga0114183_10545	2656209723	*Roseivirga seohaensis* SW-152	SAMN04423148	28077207
Ga0170448_3258	2729662814	*Ulvibacter antarcticus* DSM 23424	SAMN06264851	REFC00000000
Ga0310487_0626	2799156613	*Roseivirga ehrenbergii* DSM 102268	SAMN10864729	SMGS00000000
Ga0336340_1074	2835308220	*Winogradskyella sp.* KYW1333	SAMN09667312	QPHL00000000
Ga0336670_2825	2837407865	*Aequorivita lipolytica* CIP 107455	SAMEA4644770	30225207
Ga0336672_3534	2835103747	*Aequorivita antarctica* CIP 107457	SAMEA4644771	PMC6139392
Ga0443045_01_3096381_3097484	2884436497	*Nonlabens sp.* Ci31	SAMN12697551	CP043633
pgond44_05165	2533771942	*Psychroflexus gondwanensis* ACAM 44	SAMN02471957	24391155
Ga0077372_102327	2628626758	*Aequorivita vladivostokensis* KMM 3516	SAMN03084320	JSVU00000000
Ga0114184_10616	2654412999	*Roseivirga echinicomitans* KMM 6058	SAMN04382068	LRDB00000000.1
Ga0248413_145580	2812952679	*Roseivirga ehrenbergii* KMM 6017	SAMN03084331	LQZQ00000000
Ga0373279_1152	2830035069	*Lewinella antarctica* DSM 105096	SAMN13172327	PMC6139392
Ga0336671_791	2835108492	*Aequorivita sp.* CIP 111184	SAMEA4704834	UEFQ00000000
Ga0399719_270	2836794304	*Aequorivita sp.* H23M31	SAMN10518960	CP034951
Ga0441974_01_521424_522518	2890652824	*Ulvibacter marinus* KCTC 32322T	SAMD00166797	BKCG00000000.1
