# Supplementary figures and images for: The Bacteroidetes Aequorivita sp. and Kaistella jeonii Produce Promiscuous Esterases With PET-Hydrolyzing Activity
Source: Front Microbiol. 2022 Jan 5;12:803896. doi: 10.3389/fmicb.2021.803896 (PMC8767016; doi:10.3389/fmicb.2021.803896)

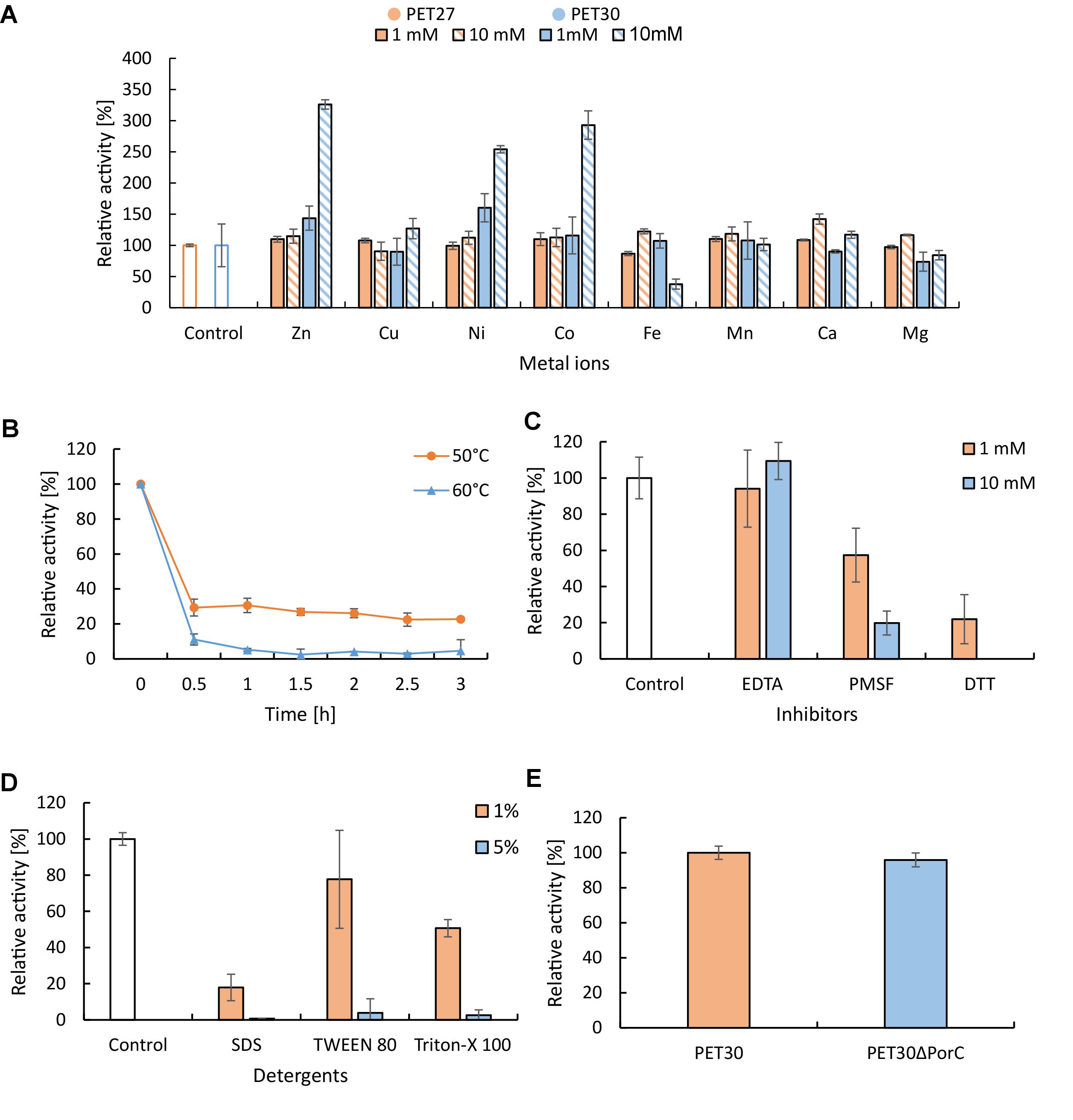

Supplement: Supplementary Figure 1 — Further biochemical characterization of PET27 and PET30 using pNP-substrates. Cofactor requirements were tested for PET27 with pNP-C8 at 40°C and for PET30 with pNP-C6 at 30°C (A). For PET30, all other tests were carried out with pNP-C6 and all assays except (B) were conducted at 30°C. Thermostability of PET30 was assessed at 50 and 60°C over 3 h with pNP-C6 (B). Inhibitors (C) and detergents (D) generally decrease the activity of PET30, particularly with higher concentrations. Activity of PET30 and PET30ΔPorC were compared under the same conditions using pNP-C6 (E). Data represent mean values of at least three independent measurements. [file Image_1.JPEG]

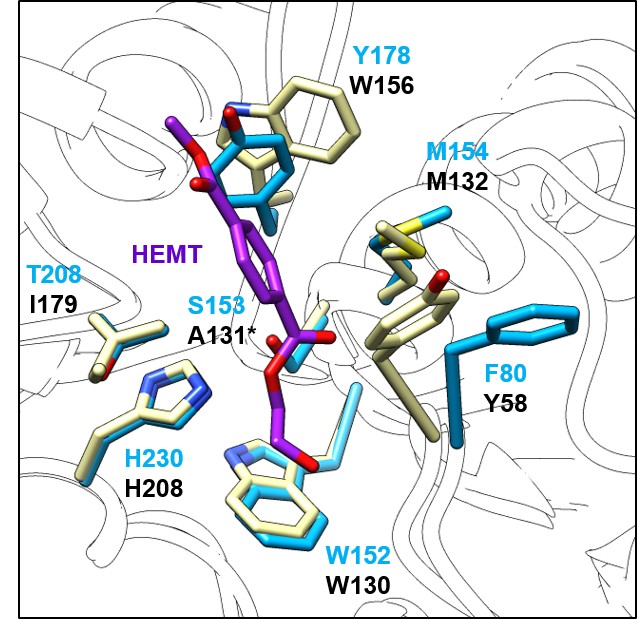

Supplement: Supplementary Figure 2 — Zoom into the active site of PET30ΔPorC. Overlaid are the structures of PET30ΔPorC (blue) with the PETase from I. sakaiensis (light yellow, black labeling) in complex with HEMT (purple; PDB code 5XH3). The residues involved in binding are highlighted and numbered according to their structure. *The structure 5XH3 is a mutant where the catalytic Serine was mutated to an Alanine. [file Image_2.JPEG]

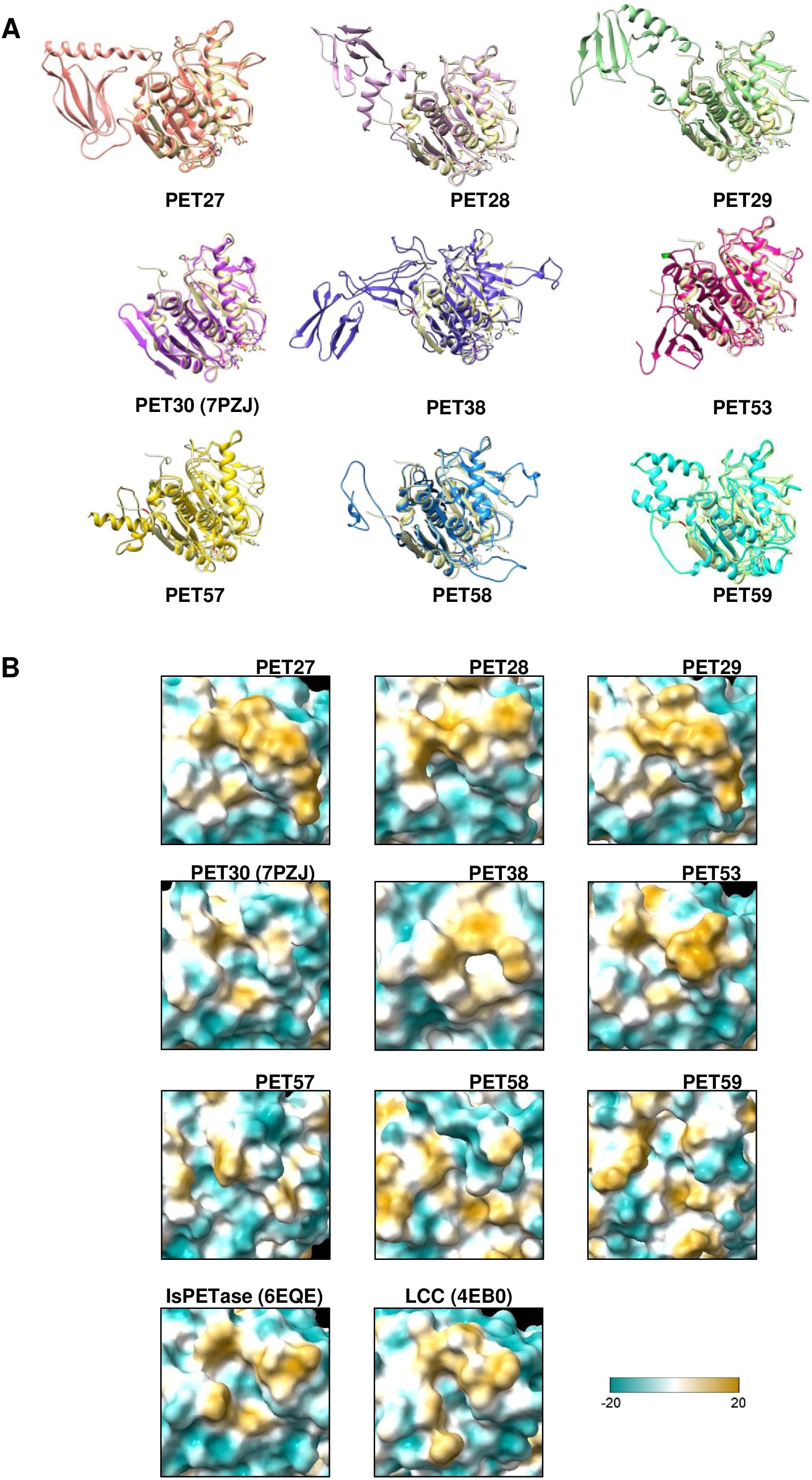

Supplement: Supplementary Figure 3 — Structure prediction models of verified and predicted PETases affiliated with the phylum of the Bacteroidetes. (A) 3D structures were modeled using the Robetta server using the IsPETase crystal structure (light yellow, 6EQE) as a backbone. For PET30, the crystal structure was shown. (B) Surface hydrophobicity around the tunnel leading to the active site of putative bacteroidetal PETases and functionally verified PET-degrading enzymes. Hydrophilic regions are displayed in turquoise and hydrophobic in gold. [file Image_3.JPEG]

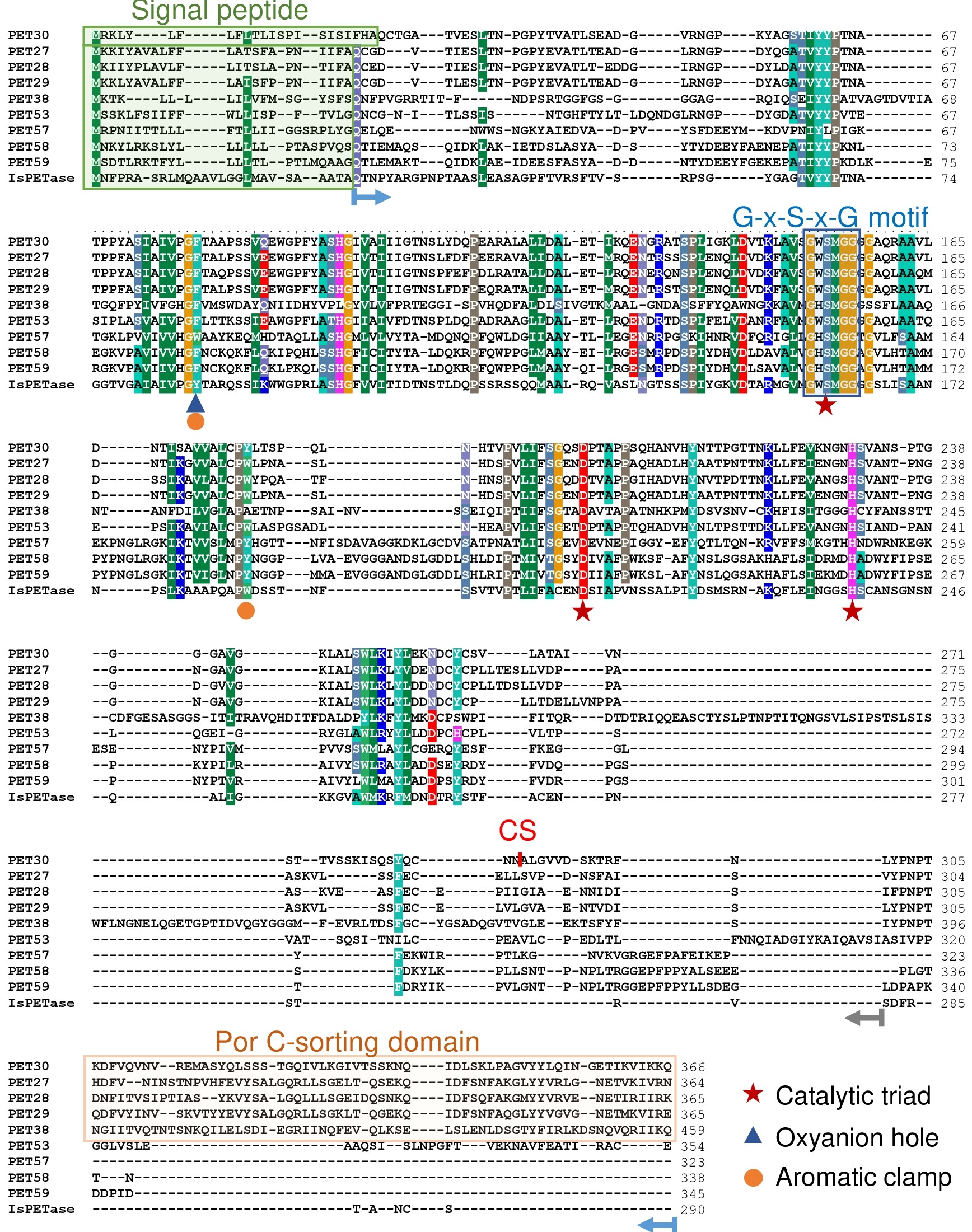

Supplement: Supplementary Figure 4 — Amino acid alignment of 9 potential PETases affiliated with the Bacteroidetes phylum. The original sequences were used for the structural alignment and the alignment was constructed with T-Coffee. Alignment was visualized with Bioedit version 7.0.5. The IsPETase was included for reasons of benchmarking. Blue arrows indicate the start and the end of the active PET27 and PET30 clones. We introduced a methionine as the first aa of the protein sequences. The signal peptide deleted version of the enzymes was functionally verified. The red arrow labeled with CS indicates the predicted cleavage site of C-terminal PorC domain (Lasica et al., 2017). The gray arrow indicates the C-terminus of the truncated version of PET30 (PET30Δ300-366), number indicates the position of amino acid). PET30Δ300-366 was active on PCL and BHET. [file Image_4.JPEG]

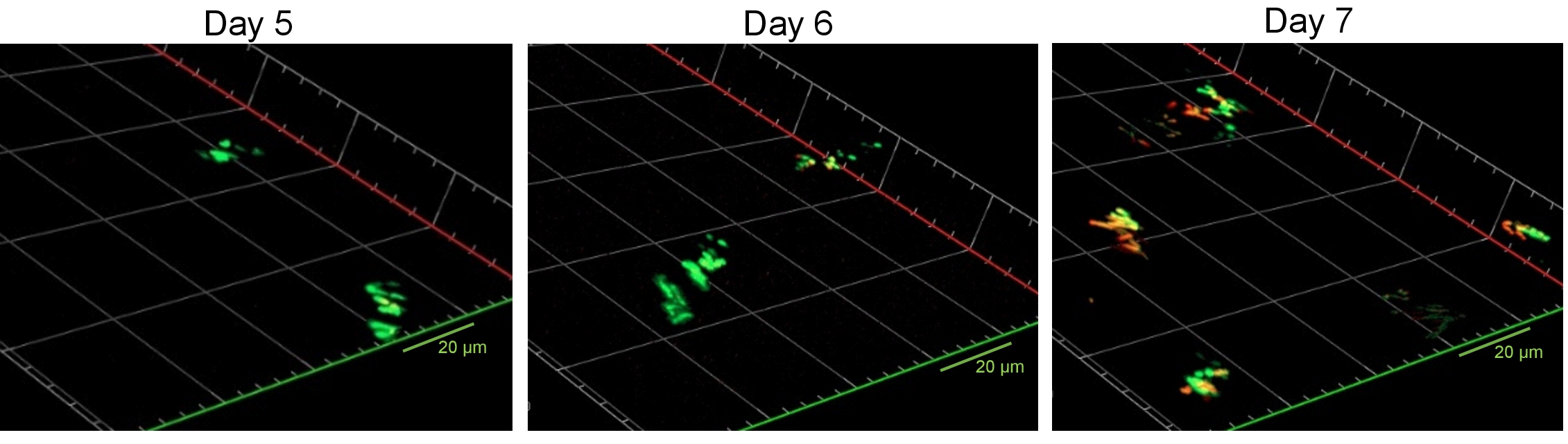

Supplement: Supplementary Figure 5 — Confocal microscopic pictures of K. jeonii colonizing PET foil. The pictures were taken after 5–7 days of incubation of K. jeonii in R2A medium. Cells are dyed with LIVE/DEAD™ stain. Green fluorescence shows living cells, red fluorescence indicates dead cells. 2D front pictures were taken with Axio Observer Z1/7, LSM 800 (Carl Zeiss, Jena, Germany) of a 3D Z-stack image. [file Image_5.JPEG]
